# Network assessment of demethylation treatment in melanoma: Differential transcriptome-methylome and antigen profile signatures

**DOI:** 10.1371/journal.pone.0206686

**Published:** 2018-11-28

**Authors:** Zhijie Jiang, Caterina Cinti, Monia Taranta, Elisabetta Mattioli, Elisa Schena, Sakshi Singh, Rimpi Khurana, Giovanna Lattanzi, Nicholas F. Tsinoremas, Enrico Capobianco

**Affiliations:** 1 Center for Computational Science, University of Miami, Miami, FL, United States of America; 2 Institute of Clinical Physiology, CNR, Siena, Italy; 3 CNR Institute of Molecular Genetics, Bologna, Italy; 4 IRCCS Rizzoli Orthopedic Institute, Bologna, Italy; 5 Endocrinology Unit, Department of Medical & Surgical Sciences, Alma Mater Studiorum University of Bologna, S Orsola-Malpighi Hospital, Bologna, Italy; 6 Department of Medicine, University of Miami, Miami, FL, United States of America; Universidade de Sao Paulo, BRAZIL

## Abstract

**Background:**

In melanoma, like in other cancers, both genetic alterations and epigenetic underlie the metastatic process. These effects are usually measured by changes in both methylome and transcriptome profiles, whose cross-correlation remains uncertain. We aimed to assess at systems scale the significance of epigenetic treatment in melanoma cells with different metastatic potential.

**Methods and findings:**

Treatment by DAC demethylation with 5-Aza-2’-deoxycytidine of two melanoma cell lines endowed with different metastatic potential, SKMEL-2 and HS294T, was performed and high-throughput coupled RNA-Seq and RRBS-Seq experiments delivered differential profiles (DiP) of both transcriptomes and methylomes. Methylation levels measured at both TSS and gene body were studied to inspect correlated patterns with wide-spectrum transcript abundance levels quantified in both protein coding and non-coding RNA (ncRNA) regions. The DiP were then mapped onto standard bio-annotation sources (pathways, biological processes) and network configurations were obtained. The prioritized associations for target identification purposes were expected to elucidate the reprogramming dynamics induced by the epigenetic therapy. The interactomic connectivity maps of each cell line were formed to support the analysis of epigenetically re-activated genes. i.e. those supposedly silenced by melanoma. In particular, modular protein interaction networks (PIN) were used, evidencing a limited number of shared annotations, with an example being MAPK13 (cascade of cellular responses evoked by extracellular stimuli). This gene is also a target associated to the PANDAR ncRNA, therapeutically relevant because of its aberrant expression observed in various cancers. Overall, the non-metastatic SKMEL-2 map reveals post-treatment re-activation of a richer pathway landscape, involving cadherins and integrins as signatures of cell adhesion and proliferation. Relatively more lncRNAs were also annotated, indicating more complex regulation patterns in view of target identification. Finally, the antigen maps matched to DiP display other differential signatures with respect to the metastatic potential of the cell lines. In particular, as demethylated melanomas show connected targets that grow with the increased metastatic potential, also the potential target actionability seems to depend to some degree on the metastatic state. However, caution is required when assessing the direct influence of re-activated genes over the identified targets. In light of the stronger treatment effects observed in non-metastatic conditions, some limitations likely refer to in silico data integration tools and resources available for the analysis of tumor antigens.

**Conclusion:**

Demethylation treatment strongly affects early melanoma progression by re-activating many genes. This evidence suggests that the efficacy of this type of therapeutic intervention is potentially high at the pre-metastatic stages. The biomarkers that can be assessed through antigens seem informative depending on the metastatic conditions, and networks help to elucidate the assessment of possible targets actionability.

## Introduction

Cancer genomics deals with a wealth of ‘signals’ and ‘marks’ routinely detected through quantitative measurements obtained at wide genome spectrum. Naturally enough, a first level of complexity involves genomics, due to the diversity of biotypes (protein coding genes, non-coding RNAs) and the computational methods that need to be specifically used to retrieve them. The expected outcome of such process is a reference “signal space’ as a product of the synergy between encoding bases, transmission mechanisms, cellular networking, all factors spanning a multitude of possible states and their communication patterns [1]. The perturbations to such systems come from factors altering the internally generated signal dynamics and enabling aberrant patterns. Measuring the induced effects at systems scale and establishing the significant impacts of such influencers from multiple perturbation sources are key steps. A first aspect is the multiscale localization, by which genomic information is heterogeneously distributed across scales (from bases to megabases), and different patterns are observable but can be measured only through different resolution-sensitive tools. Another aspect involves the role of causative, correlative and confounding factors that need rigorous assessment for inferring any valuable association between observed signals and patterns.

More complexity appears in a cancer-contextualized way, and is highly heterogeneous. Each cancer is bringing the signature of general hallmarks but also specific features. Next Generation Sequencing protocols and their ad hoc methodological pipelines have clearly indicated that a variety of molecular landscapes and profiles (expressional and mutational) exist, with melanoma as an example [2]. Additionally, the centrality of large-scale transcriptome studies and mutational approaches targeted to capture non-coding RNA evidences boosted the promised impact of epigenetic features [3]. As an example, DNA methylation has been often investigated in relation to the silencing effects exerted over gene expression levels. This type of regulation affects especially the CpG islands, as these are proximal to gene promoter regions. Generally speaking, both hyper- and hypo-methylation states are relevant to cancer conditions through chromosomal instability, re-activation of retrotransposons, and oncogenic expression levels. Tumor suppressor genes are usually silenced due to hyper-methylation affecting cell cycle, invasion and DNA repair, and involving protein coding and non-coding genomic regions [4]. However, a general hypo-methylation has also been observed in comparison with normal cells [5,6]. Additionally, the study of resistance acquisition mechanisms is increasingly observed under the epigenetic lens in view of personalized treatments. In such regards, DNA methylation inhibition (5-Aza types) is in principle emerging as a treatment option in some cancers (i.e. CRC, see [7]) due to induced activation of the immune response and co-activation of both tumor suppressors and DNA repair genes.

Melanoma is a malignant tumor of melanocytes whose incidence is increasing worldwide, therefore attracting major interest in the current research [8]. With metastasis, both genetic and epigenetic alterations become relevant. Of interest in this work is to build the coupled profile of transcriptome and methylome features from two different melanoma cell lines, i.e. two of different metastatic potential (one non-metastatic, SKMEL-2, and the other metastatic, HS294T). The main characteristic is that these melanoma cell lines are subject to the same epigenetic treatment. Since we measured both transcriptome and methylome profiles, it is natural to ask whether these are possibly correlated. This relationship is quite controversial in light of the literature results. Machine learning algorithms are designed for automatically learning tasks or functions, offering the advantage of managing heterogeneity of data and scalability of methods. For instance, they can perform automated actions within networks, directed to the discovery of differential behaviors, to the detection of anomalies, to measuring the correlation between patterns. We aim to use networks to reconcile the various experimental, computational and annotation evidences under a system’s configuration allowing the identification of melanoma targets. Ultimately, target validation may lead to understanding biological mechanisms relevant to melanoma, and to therapeutic intervention.

### The complexity of epigenetic regulation

Epigenetic modifications involve heritable and reversible changes in gene expression levels not referred to DNA sequence alterations, but rather to DNA methylation and histone modifications. DNA methylation is known to induce transcriptional inactivation or silencing in genomic regions (including non-coding). Silencing may also affect the expression levels of key transcriptional regulators, and exert cascading effects to downstream targets. Significant changes are especially expected in the presence of cancer. Hyper-methylation is considered responsible for transcriptional quiescence and suppression of expression. In turn, microsatellite instability and higher mutational frequency may be triggered. These activities are supported by the chromatin structure close to the gene promoters. By affecting DNA methylation, the transcriptional activity states get perturbed. Methylation within gene promoters can turn off their potential of suppressing tumorigenesis, cell adhesion, differentiation and growth. All such processes play roles in tumor initiation, metastasis and thus progression. Regarding progression and response to drug treatment, it is important to quantify epigenetic drivers of clonal heterogeneity [9].

Differentially methylated genes (DMG) and differentially expressed genes (DEG) can reveal cross-correlative patterns for their differential profiles, here indicated by DiP. Increased methylation levels tend to be associated with decreased expression levels at gene promoters, and such correlation seems harder to observe at gene body level [10,11]. This difference implies that reasoning in predictive terms from methylation to expression levels remains an uncertain task, mostly supported by methylated promoters in correspondence with gene silencing, and contrary to non-methylated promoters. To bypass this limitation, research focused on how networks may identify ‘epi-oncomarkers’ [12], and ‘epigenetic modules’ [13]. These ideas were used in pan-cancer studies [14]. Here, the observed correlative or causative dynamics call for further investigation, and single cancers already represent highly complex systems. For instance, melanoma involves multiple signaling pathways, cell cycle regulators and apoptotic control mechanisms whose dynamics can be studied at a genomic network scale [15]. Interestingly, while the genetic predisposition explains no more than 10% of melanoma cases, and BRAF is the most frequently mutated gene (in up to 70% of cases), the role of epigenetics and epigenetically driven regulatory networks in both initiation and progression of melanoma remain to be clarified. This holds with regard to the associations between epigenetic marks and non-coding RNAs, and the co-regulations or mediations thus induced [16].

### Melanoma cell state transitions

Despite primary tumors cause widespread cells dissemination, only a relatively limited fraction of them form metastasis in the end [17]. Cell adhesion mechanisms are crucially involved in basic cellular processes and their changes are implicated in cancer through the loss of control of cell proliferation and the start of metastatic dynamics. Changes in cell adhesive properties specifically induce plasticity, which plays a central role for metastatic phenotype development. *Cadherins* and *Integrins* are examples of adhesion molecules. *Cadherins* [18] exert functions at intercellular level, mainly communicating by cell connections through calcium ion-dependent binding. Instead, at an intracellular level, *cadherins* bind to catenin molecules establishing links with the cytoskeleton. Their specific role in melanoma is illustrated, among other references, in [19]. Their role in tumor progression is illustrated in [20]. Correlation between reduced levels of E-type cadherin and reduced survival has been studied recently in various melanoma types [21]. Here, it was also investigated in vitro transcription restoration following *5-aza-dC* treatment (DNMT1 silencing), indicating an inverse correlation with a potential therapeutic role for promoter demethylation re-activating cadherin expression.

With regards to the role of *integrins*, and their effects on migration and invasion potential, it is known that melanoma cell lines with different metastatic power exhibit heterogeneity [22]. In general, *integrins* function like ‘radars’, i.e. transmembrane cell-surface receptors, by bridging between the extracellular matrix and the cytoskeleton when environmental changes are detected. They are involved in multiple key processes, such as differentiation, adhesion, migration, proliferation and survival [23]. Finally, the interplay between *integrins* and growth factors presents clear opportunities for therapeutic targeting [24].

A study showed that BRAF (V600E) is associated with specific methylation changes, thus driving effects over target genes and activating growth signaling [25]. RASSF1A is another example of tumor suppressor gene involved in cell cycle and apoptosis, and representing a good candidate marker of tumor progression. Furthermore, it often results hyper-methylated, while deregulated in expression levels. Re-expression can be induced by *5-aza* through demethylation of its promoter. Another study focused on the reprogramming phase between proliferative (SOX10/MITF) and invasive (AP-1/TEAD) melanoma cells (from biopsies) through the integration between transcriptomic and epigenetic modifications and methylation profiles [26]. These last authors inferred a functional network representing cell state transition, probably driven by the tumor microenvironment, and explaining the observed transcriptional reprogramming. It is important to stress that changes in network dynamics may facilitate the identification of cancer pre-transition states. These may not necessarily occur at a global network scale, which remains difficult to detect and interpret, but preferably at a sub-network interactomic or modular scale, which can be algorithmically computed and evaluated in its significance [27–29].

In order to understand differential methylation patterns, also CpG island tracts are usefully studied, as from the exploration of coding and non-coding regions in cell lines, patient samples, melanocytes of various types [30]. Here, early and late stage melanoma differentiated in retrotransposable elements and CpG island signatures, indicating methylation-driven progression. Also, a co-expression network was built to establish the relationships between metastasis-linked genes subject to 5-aza treatment. An earlier study [31] pointed out gene expression profiles differences between primary and metastatic melanoma in an attempt to identify a progression-linked transition point. Specific gene sets appeared associated to the two conditions, and a transition period indicated a signature of the metastatic phenotype emergence (including oncogenes and suppressors). Asymmetry in expression was also found in another study through RNA-Seq of 3 distinctly pathogenic cell lines (normal, onset and metastasis) [32]. A special discriminatory role was here assigned to cytokine regulatory pathways, together with differential networks (cell death, cell cycle, cellular development).

### Rationale of the proposed study

Our study aims to verify significant systemic effects of epigenetic treatment while informing on the influence exerted by melanoma metastatic states. All the significant evidences obtained from transcriptome and methylome profiles where mapped at the interactome scale, a natural ground for assessing intricate regulations and identifying possible targets. In such regards, networks applied to large-scale ‘panomics’ have contributed to elucidate a series of hypotheses, such as identifying phenotypic drivers in targeted therapies (precision medicine approach) or the applicability of a reproducible cycle based on models, validations, and therapies acting inter-connectedly as a system (system medicine approach) [33]. Our use of the interactomes is aimed to the drawing of reference differential maps of coupled transcriptome-methylome DiP informative of the metastatic influences.

Concerning such regulations, the final part of our work is dedicated to melanoma associated antigens, and we use network inference in this context too. Several results have recently appeared showing T cell specific to neoantigens in cancer patients in view of developing cancer vaccines [34]. For instance, it is known that an immune response can be induced in melanoma patients, and thus melanoma cells express antigens as targets for immunotherapy [35,36]. These antigens are considered immune-activating and can provide diagnostic and prognostic utility. Melanoma has a quite hyper-mutated genome which increases the chances of neo-epitope formation [37]. This in turn explains why melanoma remains an excellent candidate for targeted immunotherapy. Several studies have considered somatic mutations for achieving predictive signatures associated with neo-antigen loads [38]. Antigens heterogeneity and profile stability are subject to variation depending on tumor, and especially cell proliferation stages [39]. It appears that T cell immunity acting against tumor-driven amino acid substitutions in melanoma patients presents a great potential outsourcing of antigens, which in turn requires systems analysis in order to identify targets of anticancer immunity.

## Materials & methods

### Cell lines characteristics

SKMEL-2 (ATCC HTB-68) is a cell line derived from patient skin malignant melanoma cells, and is known to express wild-type BRAF and mutant NRAS. HS294T (ATCC HTB-140) is a cell line derived from patient lymph node metastatic site, and has an enhanced proliferative rate. In terms of cell culture and treatment, SKMEL-2 and HS294T were purchased by American Type Culture Collection (ATCC) (Rockville, MD) and cultured under recommended conditions. Both human melanoma cells were maintained in Dulbecco's Modified Eagle's Medium (DMEM) supplemented with 10% Fetal Bovine Serum, 2 mM L-glutamine and 1% penicillin-streptomycin, at split ratio of 1:3 twice a week and incubated at 37°C in a 5% CO2 air atmosphere. For treatments, cells were seeded in 6-well plates at a density of 5 x 10^4^ cells. After 24 hours from splitting, the culture medium was replaced with media containing no drug (control) or 10 μM 5-AZA-2’-DEOXYCYTIDINE (DAC) (treated cells) (Sigma- Aldrich) for 72 hours.

### Cell cycle analysis

**Fig 1:**
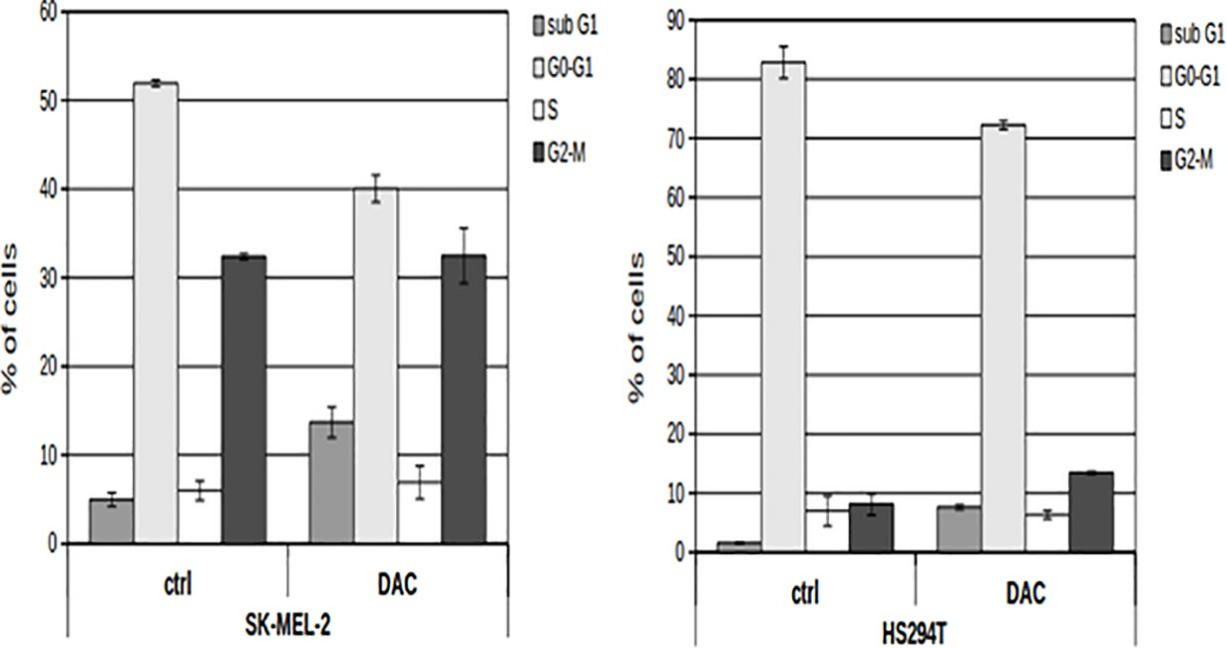
Analysis of cell cycle phases of SK-MEL-2 (left) and HS294T (right) for both untreated (ctrl) and treated (DAC) cells with 10 μM DAC for 72h. The cells % in sub-G1 (apoptotic cells), G0-G1, S and G2-M cell cycle phases are reported in Y axis. Data are reported as mean ± sd. Statistical significance threshold set at p-value < 0.05.

After 72 hours of incubation with the culture medium containing (or not) 10 μM DAC, control and treated cells were harvested and their cell cycle phases were analysed by flow cytometry (FACS). For analysis, nuclei were stained with 10 μg/ml propidium iodide (PI) in hypotonic solution (1X PBS containing 0.1% sodium citrate and 0.1% Triton X-100) for 30 minutes at 4°C in the dark. Cell cycle phases were analyzed by a FACS-Canto-II flow cytometer (BD Biosciences, San Jose, CA, USA) and data were analyzed with the FlowJo (Ashland, OR, USA) software. To evaluate the effect of DAC treatment on both melanoma cells, a one-way ANOVA was performed (cell cycle phases of treated vs control). Statistical significance threshold was set at a p-value < 0.05. The efficacy of different DAC concentrations administered at different time points to both melanoma cell lines was tested and following each treatment, and the effect on cell cycle was monitored by flow cytometric analysis. Fig 1 shows that treating both cell lines with 10μM DAC for 72h has induced a significant enrichment in sub-G1 peak (one-way ANOVA P < 0.05) in comparison to untreated cells. Since population in sub-G1 peak contains dying cells (apoptosis), it is an indicator of drug effect on cell viability.

### DNA and RNA extraction

Genomic DNA was extracted from untreated SKMEL-2 and HS294T cells, using QIAamp DNA mini kit according to the manufacturer's instructions. DNA concentration was determined by NanoDrop spectrophotometer (Thermo Scientific). DNA integrity was checked by 1% agarose gel containing 1 μg/ml ethidium bromide. Fresh prepared DNA samples were sent to BGI TECH SOLUTIONS (Hong Kong) for methtylation analysis by Reduced Representation Bisulfite Sequencing (RRBS). Total RNAs were extracted from both treated and untreated melanoma cells using NucleoSpin RNA isolation kit (Macherey-Nagel) according to the manufacturer's instructions. RNA concentration and purity was determined by NanoDrop spectrophotometer (Thermo Scientific). Fresh prepared RNA samples were sent to BGI TECH SOLUTIONS (HongKong) for transcriptome sequencing by RNA-Seq.

### RNA-Seq

The sequenced data were processed for DEG and ncRNA profiling using tools from the *R* environment. We used read counts to assess genomic coverage, and when less than 10 mapped reads were counted, the call was discarded. The read counts were then normalized by the *edgeR* package in Bioconductor [40]. The read counts were assessed for treatment and control samples under the negative binomial distributional assumptions. Differential expression in *edgeR* is assessed gene-wise by a Fisher’s exact test adapted to handle data over-dispersion. Variation estimates were performed by choosing a *Generalized Linear Model* (GLM) to account for gene-specific biological variation. Genes with |logFC|>1x and FDR<5% were established as DEGs. In choosing among various alternative models, the exact Test was found to generate less DEGs than GLM, actually providing subsets of those obtained with GLM. Hence, we selected GLM results (S1 Tables include DEGs).

The differential values were detected in non-coding genomic regions too, and involved ncRNA categories further classified into major biotypes, such as lincRNA, pseudogenes, antisense, among all other biotypes that were found. This classification (S2 Tables) was obtained by using the Ensembl genome annotation (*Homo_sapiens*.*GRCh38*.*85*.*gtf*) as a guideline for annotation (S3 Tables). The parental genes associated with pseudogenes were determined by an in-house resource of pseudogenes-parental genes associations. The criteria for assessing transcribed evidence of significant associations are: 1) Reads mapped to the pseudogene sequence and not to the parental gene; 2) Reads mapped to both the pseudogene and to the parental gene, but with lower sequence similarity (<90%). Target parental genes were identified by aligning the pseudogene sequences using BLAST against a database of the protein coding CDNA sequences from Ensembl (v. 72). The best hit matches for a pseudogene sequence were selected based on e-value scores, and the best overall hit for a pseudogene was selected as its parental gene.

The contextual analysis of lincRNAs with respect to the DEG targets was established on the basis of the physical proximal distance at chromosome level. The lncRNAs are generally classified into cis- and trans-regulatory biotypes, influencing how they may target proteins and then determine RNA–protein interaction. One looks at whether the lncRNA regulates neighboring genes, i.e. genes on the same chromosomal regions where they are located, or instead distant genes, i.e. on other chromosomes. Notably, lncRNAs interact directly or indirectly within genomic regions, sometimes through proteins performing specific biological functions, sometimes through other neighbor lncRNAs in a coordinated way. In our application, locations of DE lincRNAs were obtained by simple steps: a) lincRNA at their genomic locations (start and end positions) and b) protein-coding genomic locations (start and end positions); c) lincRNA neighbors annotation. Genes at both left and right sides of starting and ending positions of lincRNAs, and within various intervals, say ±1, ±2, ±3 Mbps regions, were considered as putative targets. Despite these intervals remain arbitrary, there is not a universally accepted definition of an accurate range. In an attempt to explore neighbors of the lncRNA locus that confidently allow putative targets to be identified, we assigned priority to targets located at the closest possible distance from the locus of interest.

As per the correlation between methylation on promoter region and gene expression, DEGs with |logFC| greater than 1.2x and methylation level on either promoter region or gene body greater than 0 were selected for performing correlation analysis. The methylation levels and expression values (FPKM) of treatment were normalized between 0 and 1. The linear model among expression of treated, methylation level on gene body and promoter region were accessed by *lm* (formula = treated ~ promoter + gene) and *gls* (model = treated ~ promoter + gene) functions in the *R* package. In both models, methylation level at promoter is significantly correlated with expression of treated (p<5%) while methylation level at gene body show no sign of correlation with expression (S1 Text). Extensive details on cross-profiles (transcriptome-methylome) are reported in S4 Tables and S5 Tables.

### Bioannotations

Gene annotations (S6 Tables and S7 Tables) were retrieved from *DAVID 6*.*8* (Database for annotation, Visualization and Integrated Discovery) (https://david.ncifcrf.gov/), an integrated database of data and tools like ENSEMBL, NCBI, UniProt, KEGG, BIOCARTA, PANTHER, BIND, GO and PUBMED etc.

Following RNA-Seq, the identified ncRNAs were cross-checked with the *lincRNom*e db (http://www.lncrnablog.com/tag/lincrnome/) and the *lncrnadb* (http://www.lncrnadb.org/) to understand the functionality details (S2 Text). From the *DAnCER* db (http://wodaklab.org/dancer/) (Disease Annotated Chromatin Epigenetics Resource) [41] Recurring genes involved in chromatin modeling were also checked (S3 Text), and bio-annotations (molecular function and biological processes) were integrated also by *BiNGO* (http://apps.cytoscape.org/apps/bingo) [42].

### Networks tools

Networks were generated from the *STRING db* (https://string-db.org/) by importing as sources the DEG and DMG (primarily, but not only, up-methylated) lists. The annotations reported on the maps have been obtained by the internal knowledgebase, and cross-checked within the *GeneCards* system (http://www.genecards.org/). The ncRNAs listed in the maps were manually curated and superimposed to the maps, after considering the previously computed associations.

### Biological validations

Drug administration for western blot analysis was performed in both HS294T and SKMel-2 by administering 10 μM 5-Aza-2'-Deoxycytidine (DAC) in culture medium for 72 hours. Western blot analysis was performed: cells were lysed in buffer containing 20 mM TRIS-HCl (pH = 7.5), 1% SDS, 1 mM Na3VO4, 1 mM PMSF, 5% beta-mercaptoethanol and protease inhibitors. After sonication, centrifugation and protein quantification by Bradford method, 15micrograms of proteins were subjected to SDS gradient gel (5–20%) electrophoresis and transferred into a nitrocellulose membrane. Incubation with primary antibodies: anti-PARP1 (Santa Cruz, sc-7150), anti-lamin A (Abcam ab26300) and anti-GAPDH (Millipore) was performed overnight at 4°C. Secondary antibodies were applied for 20 minutes and immunoblotted bands were revealed by Invitrogen ECL detection system. Immunoblotted bands were analysed by a BioRad Densitometer. Then, statistical analysis was performed from data over three independent experiments and using the Student's t-test. Data were reported as mean values with standard deviation (statistical significance values were associated to symbols as follows: *: p<0.05; **: p<0.01; ***: p<0.001).

## Results

### Detections

**Fig 2:**
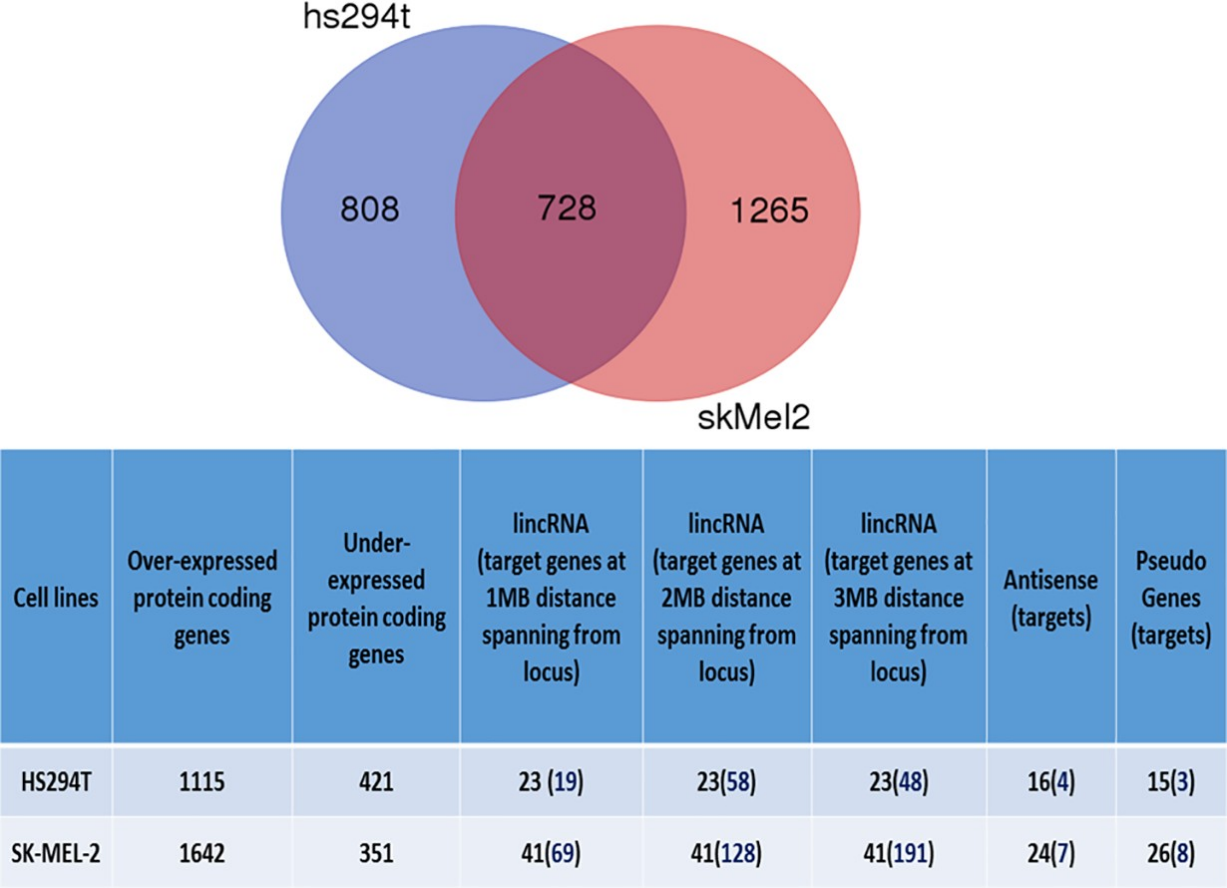
Venn diagram of DE biotypes (top). Cell lines detections by biotype.

Both protein coding genes and non-coding RNA expression values were considered components of interest for the DiP obtainable from each cell line by performing RNA-Seq. Table 1 summarizes these biological entities (details in S1 Tables). Fig 2 lists together with the detections found in common between cell lines (Venn diagram), the genes considered putative targets associated to a) lincRNAs, and retrieved within the flanking region of 1MB upstream to start position and downstream to end position; b) antisense; c) pseudogenes, considering their parental genes. Note that some of the antisense, pseudogenes and lincRNAs were also identified as part of the DiP at the transcriptome level. Also note that metastatic values were half distinctly detected and half shared with the DiP found in the non-metastatic cell line. Table 2 shows the various biotypes observed at transcript level and subsequently annotated. Table 3 shows annotations obtained by both *DAVID* and *BiNGO*.

**Table 1:** The number of differentially expressed biotypes in HS294T and SKMEL-2 cell lines.

Cell lines	Total n. of differentially expressed biotypes		Cell-specific differentially expressed biotypes
**HS294T**	**1536**		**808**
**SKMEL-2**	**1993**		**1265**
*Number of* *shared* *differentially expressed biotypes*		728	

Note: The DE biotypes were selected based on criteria involving both expression and methylation levels in the treatment group, i.e. threshold of logFC > 50% quartile of logFC values, and hyper-methylation at the promoter of unregulated DE values.

**Table 2:** Decomposition of Differentially expressed biotypes in HS294T and SKMEL-2 cell lines.

Hs294T cell line		SKMEL-2 cell line	
*Biotypes*	*GLM edgeR*	*Biotypes*	*GLM edgeR*
NA	35	NA	71
antisense	17	TEC	1
lincRNA	22	antisense	30
pseudogene	15	lincRNA	38
processed_transcript	7	misc_RNA	1
protein_coding	1435	pseudogene	26
rRNA	1	processed_transcript	12
sense_overlapping	4	protein_coding	1810
		scRNA	1
		sense_intronic	1
		sense_overlapping	1
		snRNA	1

Note: Outcome of Generalized linear models (GLM) selected in the *edgeR* function.

**Table 3:** Molecular functions annotated for both cell lines.

		source: *DAVID*		source: *BiNGO*	
Cell line N. of detections		*Statistically significant* (p-values <0.05)	*Statistically significant (**exclusive**)* (p-values <0.05)	*Statistically significant* (p-values <0.05)	*Statistically significant (**exclusive**)* (p-values <0.05)
**HS294T**					
**66**		**51**	**12**	**29**	**9**
**SKMEL-2**					
**76**		**59**	**21**	**48**	**28**
*Common* *functions*	**51**	**38**	**20**		

Note: details provided in S6 Tables and S7 Tables.

### Bio-annotations

The biological annotations (see S6 Tables and S7 Tables) are summarized next. In SKMEL-2 the KEGG pathways point to the top-5 enriched terms that include: *cell adhesion molecules* (fdr = 6.59E-04; 35 genes); *ECM-receptor integration* (fdr = 0.002231; 25 genes); *PI3K-Akt signaling* (fdr = 0.006324; 61 genes); *focal adhesion* (fdr = 0.007964; 42 genes); *leukocyte transendothelial migration* (fdr = 0.024198; 28 genes). Other pathways have been enriched with reduced significance, as with *proteoglycans*, *platelet activation*, *cytokine-cytokine receptor interaction*, *Rap1 signaling*, *and calcium signaling*. In terms of molecular functions, the most enriched terms are *extracellular space* and *region* (fdr = 6.52E-17, fdr = 2.88E-07, respectively) with 230 and 225 genes, respectively, and *plasma membrane* (fdr = 7.73E-09) with 498 genes.

Cell adhesive properties refer to tumor plasticity and play a central role in the metastatic process. They involve morphological properties and integrin expression, but also migration and invasion potential signs. Metastasis drivers act in different directions. First, the ECM-receptor integration plays a key role during tumor progression through cross-talks between cancer spots and its surrounding regions. Second, joint with the local microenvironment (niche) that determines cancer development and in response to ECM signals, also PI3K-Akt regulates the cell cycle and deals with intracellular signaling, thus affecting cell metabolism, proliferation, growth, survival and angiogenesis.

With HS294T the annotation evidenced the following terms: *cytokine-cytokine receptor interaction* (fdr = 0.01022), *NF-kappa B signaling* (fdr = 0.044757), *leukocyte transendothelial migration* (fdr = 0.048354), these all appearing with acceptable enrichment scores. In terms of molecular functions, the dominant one is *extracellular region* with 157 genes (fdr = 6.63E-18), followed by *cell differentiation* with 201 genes (fdr = 3.19E-08), *cell communication* with 143 genes (fdr = 9.82E-06), *cell motility* with 132 genes (fdr = 5.05E-05), *cytoskeletal proteins* with 103 genes (fdr = 5.58E-05), *cell cycle* with 104 genes (fdr = 8.39E-05), and *cell adhesion* with 95 genes (fdr = 1.94E-04). A major role is played at the signaling level by immunological and inflammatory factors acting in response to disease and stressors through the binding to specific receptors of target cells. These factors typically involve cytokines, chemokines and adhesion molecules, and also leukocyte transmigration.

### Chromatin remodeling

By using *DAnCER*, forty DEGs were matched with the genes involved in chromatin in HS294T. Twentytwo of them were highly over-expressed, for instance H2AFB2, CTCFL, SYCP3, AURKC, HIST1H2BG, HIST1H3D, HIST1H2BJ, SERPIND, and three were highly down-expressed, namely ZEB2, MGAM, LMNB1. Beyond being involved in DNA packaging and chromatin modelling, these genes play a role in cancer, for example the SERPIN family is involved in metastasis of cancer. Among the down-expressed genes, LMNB1 regulates PAK-2p34 by protease mediated degradation, MGAM is involved in metabolism and immune system, ZEB2 is involved in the TGF-β signalling pathway. With SKMEL-2, thirtyfour DEGs were present also in *DAnCER*, of which nineteen genes overlapped with the HS294T cell-line. Instead, fifteen DEGs were exclusively found in SKMEL-2, examples being SIRT7, PCSK4, H1F0, BCL6, SAP30, HIST1H3D, TLE4 etc. In particular, SIRT7 is involved in aging, cancer and circadian rhythm, while BCL6 is a transcriptional repressor involved in the immune system.

Among the genes considered putatively involved in chromatin remodeling, two were present as down-expressed among our DEGs, POU3F2 (transcription factor, logFC = –1.549) classified as a melanoma gene, and IRF4 (interferon, logFC = -1.833) classified as a skin neoplasma gene. Other two genes were matched, but with smaller negative expression, SOX10 (ERK signaling) and GLI2 (Wnt). With SKMEL-2 other matches were found, for instance NOS3 (logFC = 5.174), mediating VEGF-induced angiogenesis, ACTA2 (logFC = 4.862), an actin involved in cell motility. And GLI1 (logFC = 2.036), a member of the Kruppel family of zinc finger proteins encoding a transcription factor activated by the Hedgehog cascade and regulating stem cell proliferation and also p53 (negatively).

### ncRNAs, DEG targets, and a few specific annotations

Considering the identified DE lincRNAs in SKMEL-2, the targets at 1-to-3MB distance from the genomic locus were identified. There are sixtynine targets, and some deserve attention. For instance, the over-expressed KLHDC8A, providing an alternative pathway for tumors for maintaining aggressiveness in the absence of epidermal GFR dependence. Then MZB1, which causes cell-specific regulation of apoptosis, among other functions, likewise TNFRSF25 and MX2 (interferon), both important for apoptosis. When looking at the matches of our detections within the *lncRNome db* [43] (see S6 Tables), only LINC00337 was found, among those with target genes within the 1Mb of distance from locus. There are other seven target genes, namely KCNAB2, CHD5, GPR153, HES2, ESPN, TNFRSF25, PLEKHG5, and six are over-expressed and only KCNAB2 is down-expressed. Of these, CHD5 is a potential tumor suppressor regulating the expression of genes involved in cell proliferation and differentiation. Downstream activated genes may include CDKN2A, which positively regulates the p53/TP53 pathway, which in turn, prevents cell proliferation. GPR153 and HES2 are involved in transcription activity, while TNRFSF25 induces apoptosis.

Highly differential expressions of ncRNA targets appeared in relation with extracellular region functions. Considering HS294T, three identifications are worth mentioning: the over-expressed F5 and PRAP1, involved in cell adhesion, and TNC, which is down-expressed. With regards to SKMEL-2, of interest is the identification of CD14, involved in programmed cell death. Another group of functions is relevant, and involves cell junctions, i.e. multiprotein complexes that allow the contact between neighboring cells or between cells and the extracellular matrix. Especially three identifications shared across the cell lines and highly over-expressed are especially relevant: CRB3 (cell polarity in epithelial cells, regulators of morphogenesis), XIRP1 (involved with actin), and ARC (cell morphology, migration and cytoskeletal organization). Other DEGs were instead identified as uniquely over-expressed. For instance, in HS294T these are: ESAM (endothelial cell adhesion), involved in platelet activation and immune cell transmigration; GJB4 (gap junctions), providing intracellular communication; TNS4, involved in cell migration and possibly promoting apoptosis. With SKMEL-2 other highly over-expressed identifications were uniquely found, namely: CDHR2, a tumor suppressor; PECAM1, involved in leukocyte migration, angiogenesis and integrin activation; GPER1 (G-protein coupled receptor 1); S100A1, a tumor suppressor; SLC30A3, involved in transmembrane transported activity.

For the proteinaceous extracellular matrix, SKMEL-2 presents a few highly over-expressed DEGs, such as MMP9 (cell death regulator) and WNT10A (Wnt signaling), while HS294T presents SERPINA1 (hypoxia) as over-expressed and a series of down-expressed genes, such as MMP17 (metabolism), MGP (cell differentiation), CHL1 (cell adhesion and differentiation), MMP16 (proteolysis), COL11A1 (collagen). Of interest the sign concordance across cell lines of the over-expressed FBLN2 (cell adhesion) and PTPRZ1 (cell differentiation). Among the identifications uniquely found, a few appear in SKMEL-2 with the highly overexpressed WNT1 and WISP2 (Wnt), SOST (cell communication), ADAMTS14 (proteolysis), HAPLN4, ELN and COL8A2 (cell adhesion and proliferation), while then others identifications appear in HS294T as the under-expressed cell adhesion genes (among other processes), COL15A1, FREM1, GPC6 and FLRT1.

### Differential network maps

Cross-referencing of transcriptome and methylome DiP leads to network maps as a way to select regions in which the combined influence may exert effects visible through hubs, hierarchies or modules. Since the majority of detected evidences turned into hyper-methylated over hypo-methylated genes, we focused on cancer genes undergoing inactivation most likely induced by hyper-methylation, in other words those subject to epigenetic silencing. Here, treatment via demethylation influences the re-activation of their expression levels. Being these genes involved in several pathways, an additional constraint was applied by mapping DEGs from both cell lines that were found upregulated. This implies re-activation of expression levels induced by treatment.

**Fig 3:**
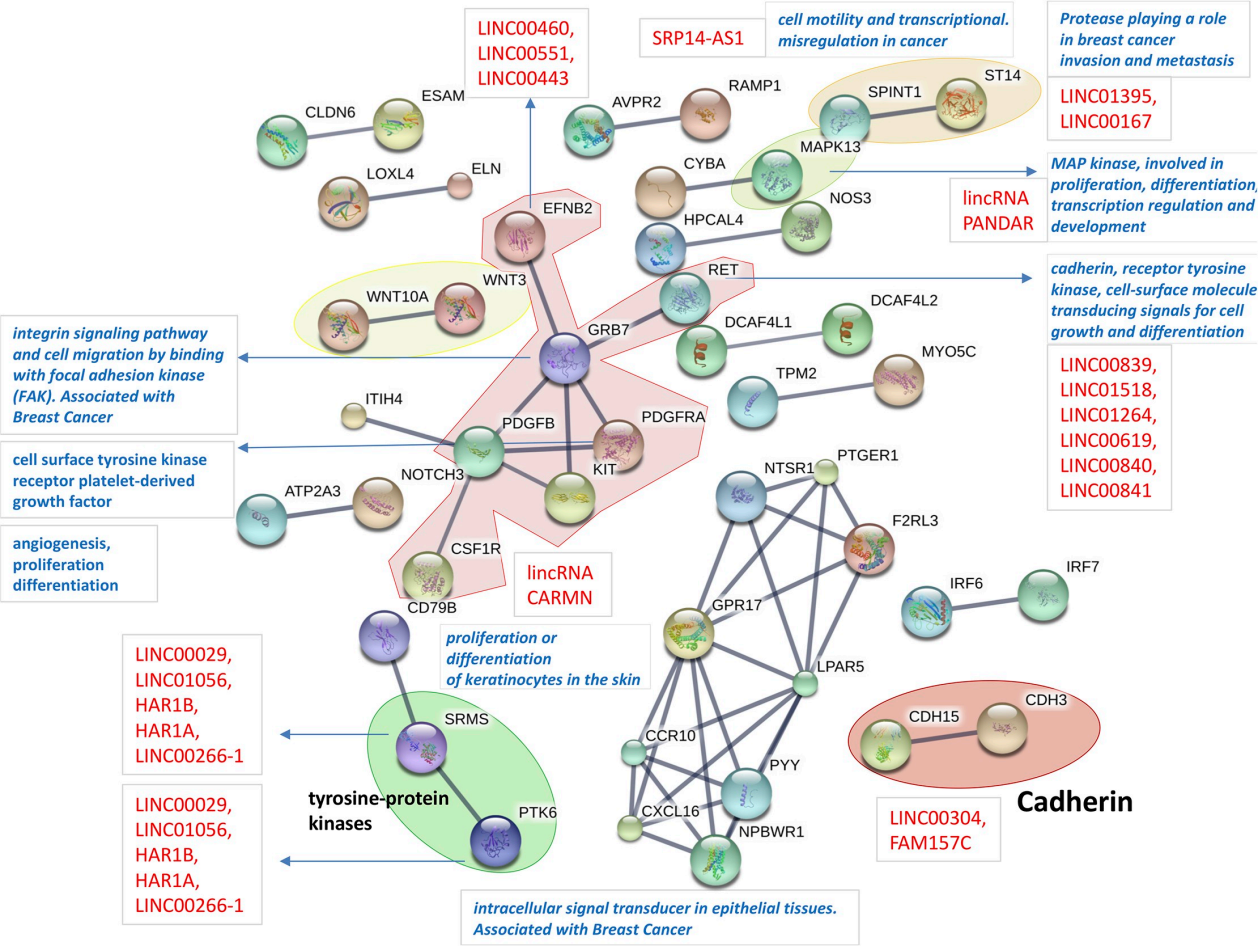
SKMEL-2 cell line map. Protein-protein Interaction map from STRINGdb obtained from DiP (DEGs and differentially methylated). The effects of treatment were analyzed in hyper-methylated DEGs to consider silencing effects on expression before treatment. The red names refer to ncRNAs, and lincRNAs depend on 1Mb distance between their locus and the associated target genes.

Looking at Fig 3, the SKMEL-2 cell line network map, a number of nodes appear annotated in various ways. In some cases these are functionally relevant (gene/module annotations recalled by blue arrows) by either themselves, as with MAPK13 (light green envelope) or NOTCH3 (involved in several functions, i.e. differentiation, proliferation, apoptosis), or because of aggregates or modules. This is the case of WNT (WNT3 and WNT10A, yellow envelope), whose role in tumorigenesis is widely known in relation with proliferation and migration, or CDH3 and CDH15 (red envelope, bottom right), i.e. cadherin of P and M type, respectively. In particular, a paralog of CDH3 is CDH1, *cadherin* of the E type, a known growth and invasion suppressor whose loss of function contributes to cancer progression through augmented proliferation, invasion, and/or metastasis, and whose mutations have been correlated with various cancers. Table 4 is next shown to indicate the cadherin values computed in the SKMEL-2 cell line. In the HS294T cell line the significance of logFC was not high and thresholds were never met at both measurements together. The observed post-treatment effect inducing over-expressions in both CDH3 and CDH15 is therefore neutralizing the loss of cell-cell dependent adhesion and influencing melanoma development and progression.

**Table 4:** Cell adhesion in view of cadherins in SKMEL-2.

SKMEL-2								
Cell line	TUMOR	TREATED	Log(FC)	Log(CPM)	Methylation		Threshold	
					Promoter	Gene Body	T > 2.24	M > 4.25
**CDH1**	0.64	5.14	2.86	3.76	3.44	21.02	T (yes)	M (No)
**CDH3**	0.28	2.09	2.72	2.35	25.99	9.28	T (yes)	M (yes)
**CDH15**	0.62	6.28	3.2	3.26	6.96	7.81	T (yes)	M (yes)
RET	0.01	0.75	7.88	0.91	4.9	3.22	T (yes)	M (yes)

**Note:** *edgeR* delivers logFC or log fold change and logCPM or log counts per million. Thresholds at transcriptome (T = 2.24) and methylome (M = 4.25) levels were computed according to quantiles (50%).

Interestingly, note that also RET (red central envelope) is marked with reference to *cadherin*, and indeed it is considered an atypical *cadherin* belonging to a group of *cadherins* endowed with a diversity of unique structures and functions but still playing a role in cell adhesion and beyond [44]. A relatively long list of lincRNAs is associated with RET. Considering Table 4 and the interaction network maps, it appears that the role of *cadherin* genes is more substantial in SKMEL-2 than in HS294T. Because the maps satisfy two constraints, namely significantly DEGs that were hyper-methylated in tumor data, at promoter and/or gene body, the effects of the epigenetic treatment over the inactivated *cadherins* is to induce a re-activation of their expression levels. This occurs in a non-metastatic context (SKMEL-2) and not in the metastatic one (HS294T). Considering this difference, and by seeing the epigenetic treatment under the lens of *cadherin* and its loss of function (cell adhesion), it appears that a functional inhibition by melanoma occurs at an early disease development stage, which is where the epigenetic treatment exerts a clear impact. The same is not observed when the disease has already progressed to a metastatic stage.

Back to RET, this gene is part of central modules (red envelope) including PDGFB and PDGFRA, which indicate platelet-derived growth factor involved in *integrin* signaling, cell migration and focal adhesion (FAK). GRB7is also part of this module, and is known to interact with *integrin* signaling, with EGFR and EFNB2 (*Ephrin* B2, a kinase being crucial for migration and adhesion), to bind with FAK, and also to communicate with the RET signaling network (see http://pathcards.genecards.org/card/ret_signaling). EFNB2 is annotated with three lincRNAs. Two WNT components are also observed (yellow module) with WNT3 and WNT10A, both implicated in oncogenesis, DNA damage and PI3K Akt signaling. A list of lincRNAs is associated with PTK6 (green module), involved in tumor growth. Finally, MAPK13 is involved in proliferation and differentiation among other processes. Of particular interest is the association with the PANDAR ncRNA, which is thought to regulate the response to DNA damage, and whose deregulation induced cancer progression. We could not find it differentially expressed among our ncRNA detections.

**Fig 4:**
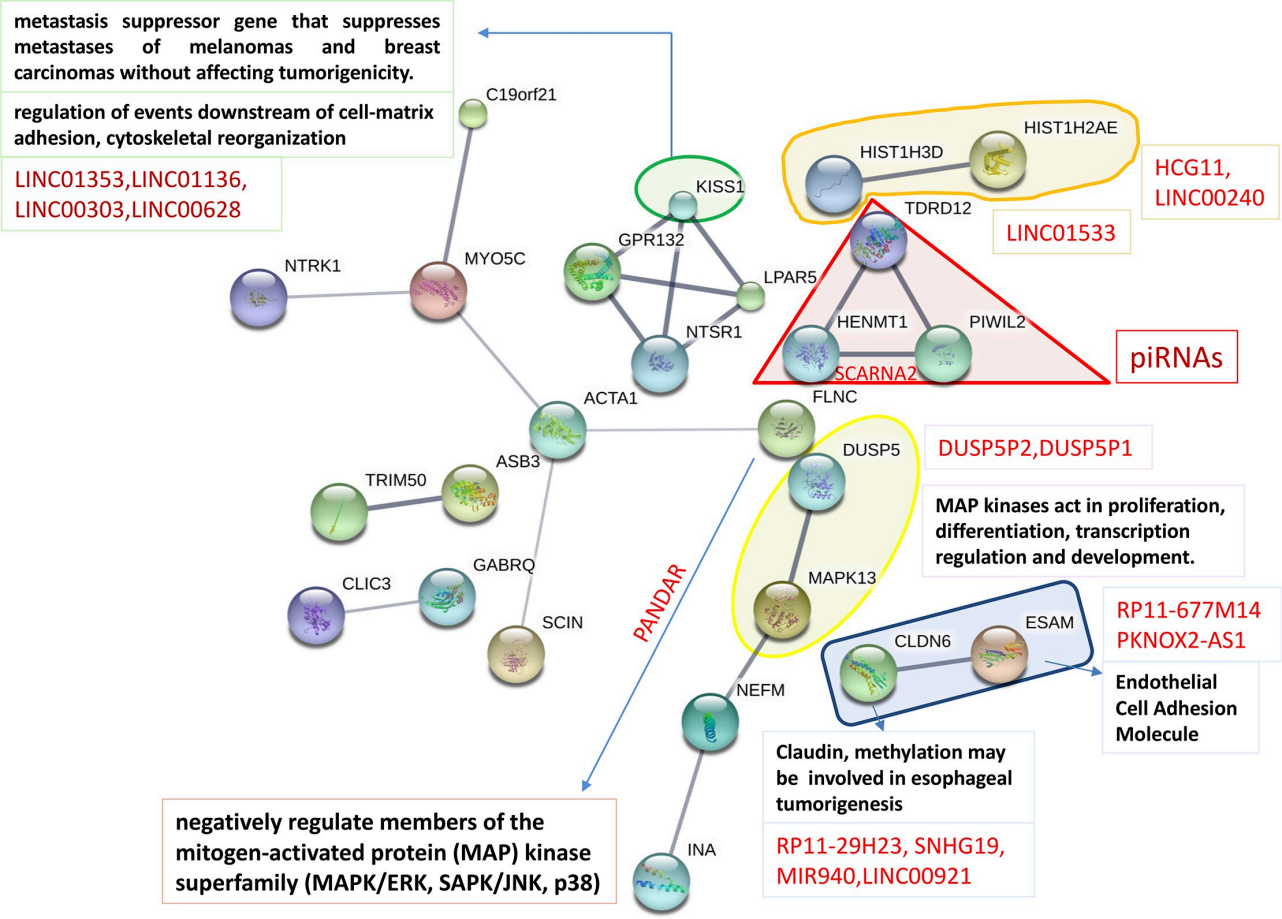
HS294T cell line map. Protein-protein Interaction map from STRINGdb obtained from DiP (DEGs and differentially methylated). The effects of treatment were analyzed in hyper-methylated DEGs to consider silencing effects on expression before treatment. The red names refer to ncRNAs, and lincRNAs depend on 1Mb distance between their locus and the associated target genes. expression before treatment.

Looking at the metastatic HS294T cell line network map (Fig 4), fewer modules appeared relatively to before. MAPK13 (yellow envelope) is annotated here too, together with the associated PANDAR and the negative regulator DUSP5. Among the different aggregates that were formed, one involves KISS1, relevant for cytoskeletal reorganization downstream cell matrix adhesion, a gene known to suppress metastases in melanoma and in some breast cancers too, by inhibiting invasion. Another gene, ESAM, i.e. endothelial cell adhesion molecule, is interacting with CLDN6 or *claudin 6* (blue module), with possible sharing of tight junction function to enable cell-to-cell adhesion. *Claudins* are examples of junctional proteins, i.e. transmembrane proteins that function to promote cell-cell adhesion, and are involved in the metastatic phenotype as both cancer promoters and tumor suppressors [45]. These proteins are natural candidates serving as therapeutic targets in cancer at metastatic stages. Associated to CLDN6 there is also a mini-list of ncRNAs, potential regulators whose functions remain largely unknown. Note that the network interactors were cross-referenced between *STRING db* knowledge base and *GeneCards*, while ncRNAs were inserted by inspecting the associations with ncRNA targets at given genomic distances from ncRNA loci. It is definitely less clear and surely less pervasive the effects induced by the demethylant with HS294T, which suggests a limited impact of the treatment in the reprogramming action against the metastatic process.

### Systems analysis of T-Antigens

**Fig 5:**
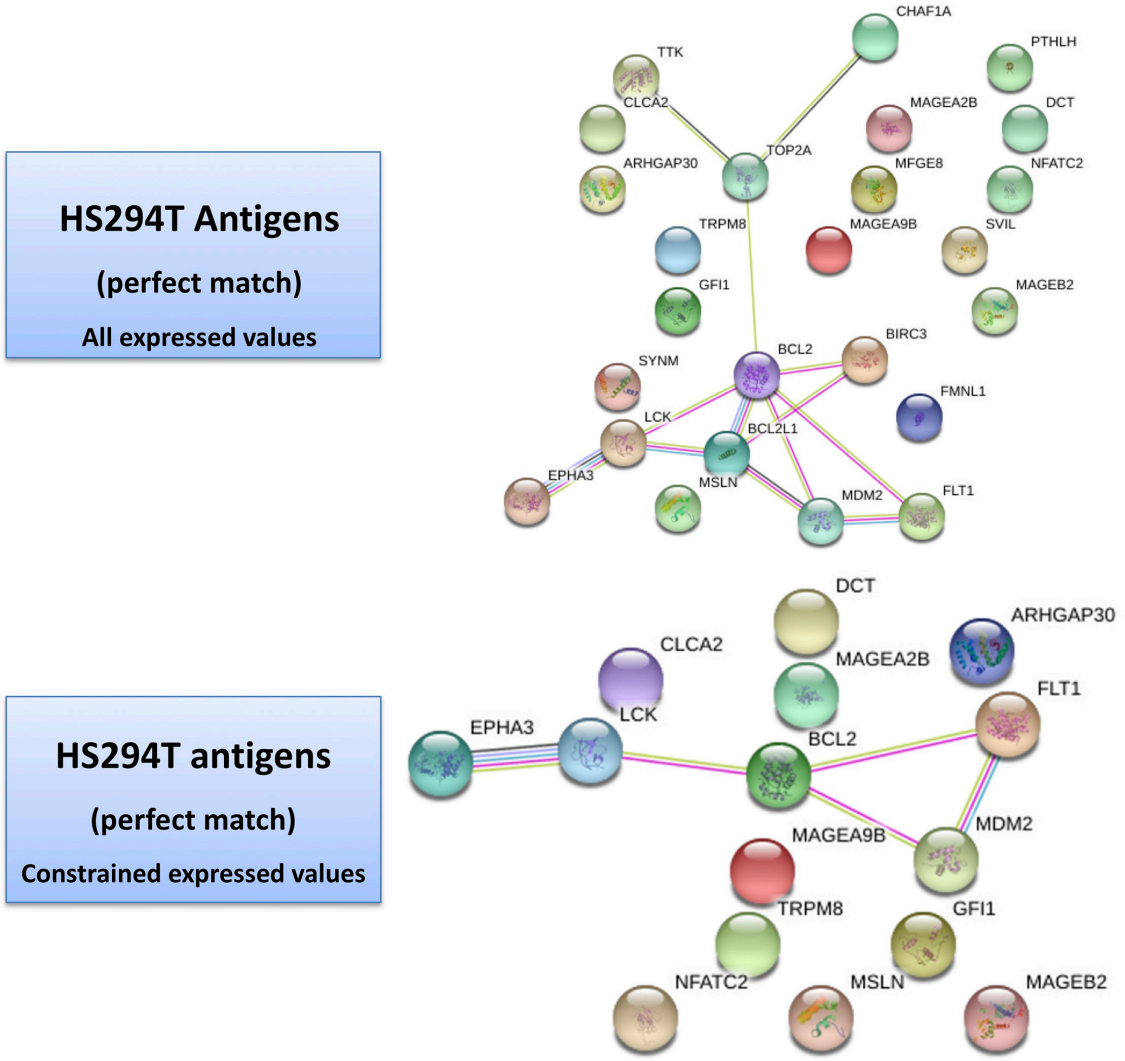
HS294T Antigen-driven map. Protein-protein interaction map from STRING db obtained from all best matched antigens (top panel)—see Table 5 and S8 Tables.

Table 5 and Table 6 refer to the list of antigens searched via the *TANTIGEN db* (Tumor T-cell Antigen Database) [46]. This is a resource on human tumor antigens based on HLA ligands, predicted binding peptides and T cell epitopes, and including reference to gene expression, isoforms, mutations. Two types of results were obtained from our profiles cross-referencing, namely the ‘best match’ and the ‘one-mismatch’ (tabulated evidences are reported in S8 Tables). Looking at the results, a few considerations follow. First, the networks establish co-expressions between nodes and/or interactions. Fig 5 for HS294T shows two panels: at the top level, one with all the gene selections with best-matched antigens; at the bottom level, one with the gene list restricted by the application of thresholds in the transcriptome (logFC>1.5, logFC<-1.5) and methylome (2.9 at promoter level, 8.39 at gene body level, as a result of 50% quintiles) values. The top unconstrained map shows the presence of various singletons, such as FMNL1 (f*ormin*-like protein 1) involved in morphogenesis, cytokinesis and cell polarity, plus members of the MAGE family of antigens (especially tumor specific proteins belonging to CTA group), whose exact functions is not completely known but appear to regulate cell cycle progression and apoptosis.

**Table 5:** HS294 antigens.

Gene name	Protein ID	Length	Antigenic peptide name	Antigenic peptide seq	Loc	LogFC	Methylation level promoter	Methylation level gene body
**BIRC3**	XP_016873132.1	604	T000939	RLQEERTCKV	550	**1.808209**		
**BCL2**	XP_011524437.1	224	T000947	NIALWMTEYL	172	**-2.10161**	1.083	**9.978**
**DCT**	XP_011519351.1	456	T000114	LLGPGRPYR	134	**-2.79781**		
**EPHA3**	XP_005264772.1	982	T000393	DVTFNIICKKCG	356	**-2.39375**		
**FLT1**	XP_016875974.1	1300	T000789	GVLLWEIFSL	1048	**-1.46441**	**7.443**	1.712
**GFl1**	XP_005270806.1	422	T001014	QPR(S)PGPDYSL[QPRSPGPDYSL]	17	**3.3185**	**18.065**	**12.974**
**LCK**	XP_011539755.1	516	T000743	DYLRSVLEDF	495	**7.500057**		
**MAGEB2**	XP_011543814.1	319	T000252	FLWGPRAYA	273	**1.8108824**		
**MDM2**	XP_005268929.1	491	T000674	YTMKEVLFYL	48	**1.815603**	0.397	**17.749**
**NFATC2**	XP_011527126.1	940	T000980	KPY(S)PLASL[KPYSPLASL]	70	**-1.81647**	**18.28**	**22.764**
**CLCA2**	XP_011540750.1	616	T000385	LLGNCLPTV	425	**2.206476**		
**MSLN**	XP_005255091.1	621	T000796	FLLFSLGWV	23	**1.457531**	3.75	4.516
**TRPM8**	XP_011510112.1	1115	T000883	GLMKYIGEV	187	**-1.61673**		
**ARHGAP30**	XP_005245127.1	1044	T001046	RPAK(S)MDSL[RPAKSMDSL]	323	**4.162052**		
**MAGEA2B**	XP_016884895.1	314	T000162	TTINYTLWR	73	**-8.76344**		
**MAGEA9B**	XP_005278249.1	315	T000196	VALELVHFLL	112	**2.124184**		

Note: values passing transcriptome and methylome thresholds.

**Table 6:** SKMEL-2 antigens.

Gene name	Protein ID	Length	Antigenic peptide name	Antigenic peptide seq	Loc	LogFC	Methylation level promoter	Methylation level gene body
**PLIN2**	XP_016869748.1	445	T000366	SVASTITGV	129	**5.632593**	**13.151**	**25.123**
**BIRC3**	XP_016873132.1	604	T000939	RLQEERTCKV	550	**2.404825**		
**FMNL1**	XP_006722125.1	1164	T000850	RLPERMTTL	799	**2.028542**	7.675	**8.02**
**GFI1**	XP_005270806.1	422	T001014	QPR(S)PGPDYSL[QPRSPGPDYSL]	17	**3.026243**	11.808	**11.394**
**LCK**	XP_011539755.1	516	T000743	DYLRSVLEDF	495	**7.499478**		
**OCA2**	XP_011519942.1	852	T000618	IMLCLIAAV	427	**-2.63585**	2.826	**13.916**
**PTHLH**	XP_011519076.1	177	T000876	FLHHLIAEI	59	**1.809974**	**7.344**	1.106
**ABCC3**	XP_005257820.1	1463	T000825	LYAWEOSFL	439	**4.109376**	0.99	5.013
**IGF2BP1**	XP_005257012.2	565	T000906	KTVNELQNL	496	**2.309944**	27.031	3.641
**SYNPO**	XP_005268427.1	903	T000979	RPSRS(S)PGL[RPSRSSPGL]	615	**1.504201**		
**RAB38**	XP_016872944.1	177	T000663	VLHWDPETV	50	**-1.22612**	0.428	0.892
**ARHGAP30**	XP_005245127.1	1044	T001046	RPAK(S)MDSL[RPAKSMDSL]	323	**1.867114**		
**MAGEA2B**	XP_016884895.1	314	T000162	TTINYTLWR	73	**8.94263**		
**CCDC88B**	XP_006718582.1	1560	T001017	SPEKAGRR(S)SL[SPEKAGRRSSL]	588	**2.423258**		
**XAGE1B**	XP_016885238.1	146	T000840	CATWKVICKSCISQTPG	98	**1.782175**		
**MAGEA9B**	XP_005278249.1	315	T000196	VALELVHFLL	112	**3.747172**		

Note: values passing transcriptome and methylome thresholds.

Of interest are the connectivity paths, and these are visible in the unconstrained map, less so in the constrained maps. In the former context, the following chain of nodes is noted: TTK, a mitotic kinase associated with cell proliferation through mitosis because critical for the regulation of cell division; TOP2A, a gene known as target for anticancer agents whose mutations are linked to drug resistance, and which encodes an enzyme altering DNA during transcription and replication; BCL2, encoding an anti-apoptotic protein (affecting lymphocytes, for instance), and the associated BCL2L1, also inhibiting cell death, and BIRC3, an inhibitor of apoptosis; MSLN, or megakaryocyte potentiating factor, involved in mesothelin generation, which is a sort of cell adhesion protein appearing overexpressed in some cancers; MDM2, which can promote tumor formation by targeting tumor suppressor proteins such as p53 and whose overexpression has been seen in various cancers; FLT1, or Fms-related Tyrosine Kinase 1, encoding a member of VEGFR of relevance to angiogenesis and vasculogenesis; LCK, of the Src family of protein tyrosin kinases and key signaling molecule involved with T-cells by binding to various cell surface receptors, also playing a key role in the T-cell antigen receptor-linked signaling transduction patwhays; EPHA3, of the ephrin receptor (part of the protein tyrosine kinases), involved in cell-cell adhesion, cytoskeletal organization and cell migration processes.

**Fig 6:**
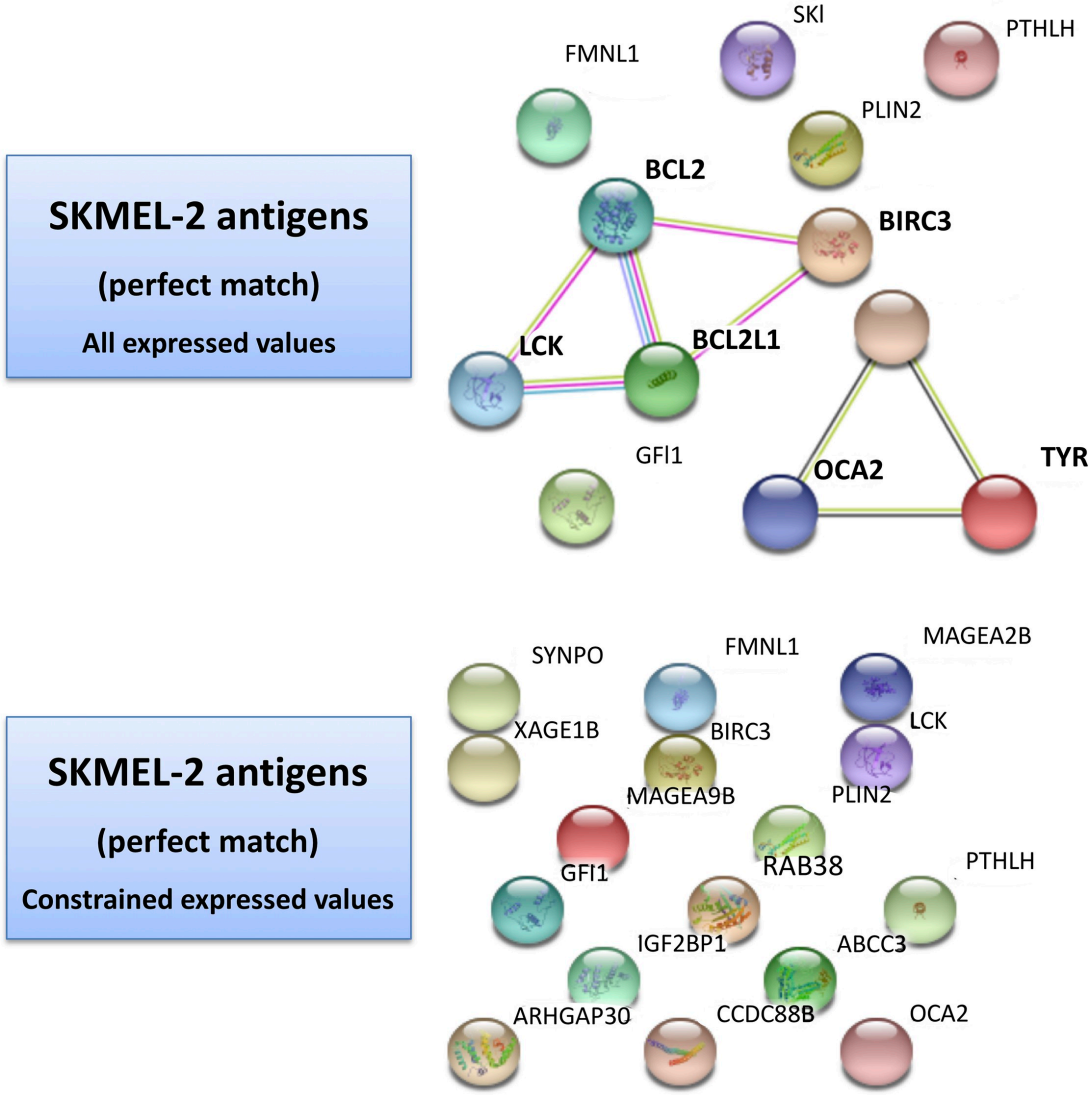
SKMEL-2 Antigen-driven map. Protein-protein interaction map from STRING db obtained from all best matched antigens (top panel)—see Table 6 and S8 Tables.

When the same analysis is performed over the SKMEL-2 map in Fig 6, much smaller connectivity is observed and the presence of singletons is dominant under constraints. The application of such constraints dissolves the connectivity observed with the entire list of genes for which the antigens were matched, and in which two paths had appeared: a) OCA2, a transmembrane protein involved in the transport of tyrosine, linked to TYR (tyrosinase) and GPR143 (tyrosine binding); b) BCL2, LCK, BCL2L1 and BIRC3. MAGE and XAGE (member of GAGE family, useful markers in melanoma and associated with poor prognosis) genes are present too.

### Biological validation

Features of programmed cell death consist of a very particular nuclear behavior involving DNA cleavage, destruction of the structural chromatin organization and proteolysis of nuclear membrane and nuclear lamina components [47]. Poly(ADP-ribose) polymerase 1 (PARP1) and Lamin A/C are cleaved in apoptotic nuclei by proteolitic enzymes, called caspases, at the late stage of apoptotic process [48, 49]. Therefore, we used these markers and tested their apoptotic cleavage by western blot analysis. Both HS294T and SKMEL-2 melanoma cells underwent apoptosis after 72 hours of treatment with 5-Aza-2'-Deoxycytidine (DAC) as indicated by cleavage of PARP1 and lamin A/C (S1 Fig). The densometric analysis indicates that PARP1 cleavage (85 kDa) was detectable in both DAC-treated cell types while significantly increasing in DAC-treated SKMEL-2, and cleaved Lamin A (45 kDa) significantly increased in both DAC- treated cell lines.

## Discussion

Gene expression profiles from melanoma cell lines may display different transcriptome states, such as of proliferative and invasive ones, which tend to switch when temporally observed and the initially observed proliferative-to-invasive state transition seems induced by chromatin-dependent transcriptional changes, among other regulatory network dynamics [25]. These states prefigure two transcriptional signatures beyond the influence of genetic mutations, as these alone are not sufficient to explain the reprogramming and also reversible dynamics, and most likely influenced by the microenvironment [50]. Mutations involved in the growth of tumor are rare but may generate aberrations (i.e. amino acid substitutions) in protein sequences, making thus the latter potential targets in view of tumor-specific immune response [51].

Our melanoma data were obtained from two cell lines, only one being metastatic. Coupled profiling was performed, and methylomes are expected to reflect characteristics that are unique to each cancer type, thus justifying differences in patterns or signatures. We hypothesize that by these signatures we might better decipher complex regulation mechanisms underlying the observed data. Transcriptomes and methylomes have been combined in many studies, but there is still not a clear understanding of how this merge can improve early diagnosis, prognosis and prediction of therapeutic response. The advantage of investigating DNA hyper-methylation versus standard tumor biomarkers is that the former can be highly relevant in predictive terms, such as monitoring the stages of cancer progression, and including early and premalignant conditions. The best example provided by our study concerns *cadherins*, in particular CDH3 and CDH15, for which a clear mark of post-treatment appears through the over-expressions of both genes. Treatment therefore contrasts the loss of cell-cell dependent adhesion underpinning melanoma development and progression, and indicating the opportunity for targeted clinical intervention similarly to what has been observed in the so-called ‘*actin*-diseases’, i.e. those with a disruption of the *E-cadherin* and *actin* connection [52].

Gene expression is known to be affected by DNA methylation. In particular, a repressive epigenetic mark is usually investigated at both promoter and gene-body located sequences. In general, methylated promoters are negatively correlated with gene expression because associated with gene silencing, while non-methylated promoters may associate independently on transcription states [53,54]. In fact, it is still to be clearly assessed whether gene body methylation levels are more or less predictive than TSS regions. In our study we noticed negatively correlated behavior between transcriptome and methylome levels at promoters, but could not observe this pattern at gene-body levels. We supported these evidences with a model employing simple statistical regressions and testing the significance for the coefficients in relation with the two profiles at both promoter and gene body level. When considering profile coverage of coding and non-coding genomic regions, correlations were assessed only at the coding regions, which is where methylome measures were obtained by reduced sequencing.

Aberrant DNA methylation affects many cancer genes, suggesting a potential use as biomarkers for early diagnosis, prognosis and also prediction of therapy response [55]. Due to the reversibility of such aberrations, there is potential for therapeutic targeting combining DNA demethylation with candidate target selection. Since hyper-methylated genes can be reactivated after treatment with methylation inhibitors, mapping these genes onto networks may elucidate possible but hard to measure correlation with differential expression. The advantage of using networks is that connected paths identified among DEGs represent a robust measure of association at a biological level. Functional epigenetic modules have been recently indicated as good instruments for integrated use with scaffold networks, especially but not only with protein networks. They are aimed at identifying hotspots, i.e. significant epigenetic dysregulation associated to key phenotypes. Examples of integrative tools are provided by *FEM* [56], *BioNet* [57] and *SMITE* [58]. Other tools have been provided without explicit use of correlated profiles (see *EMDN* [12]).

In the context of our cell lines, a feature shared by the two cell lines is MAP-kinase signaling associated with regulation of cell proliferation, something already noticed with different cancers and analyzed through network oncomarkers [12]. Several studies have pointed out the re-activation of the MAPK pathway as an effector of mechanisms of acquired resistance, and holding especially in view of MITF or SOX10 activity [59]. In our study, the modular protein interaction networks evidenced MAPK13, a target associated with the PANDAR ncRNA of relevance to therapy because aberrantly expressed across various cancers. However, since it is the diversity from metastatic levels that we are interest in, we observed the effects of demethylation under such cell-specific metastatic potential. Under non-metastatic conditions various re-activated pathways appear deeply involved in cancer hallmarks. Due to hyper-methylation induced by tumor, these pathways were altered, and the epigenetic treatment is reconfiguring them in an intricate map of network relationships. Such complex regulations include the roles of ncRNAs detected as differentially expressed and contextualized in network maps with their putative targets. Both the map configuration and the presence of ncRNAs change substantially under metastatic conditions, as the more advanced stage of melanoma progression implies a different degree of involvement of pathways. Interestingly, this emerging differentiality indicates a demethylation impact stronger at the early disease stage, most likely the best time for therapeutic intervention.

The other instruments explored in this study are the differential antigen maps, and here are a few remarks. Tumor antigens refer to tumor molecules interactive with the immune system, being some specific (not present in normal tissue) and some associated (overexpressed in tumor compared to normal) [46]. These types can be recognized by T cells and present on the cell surface by human by so-called HLA or human leukocyte antigen molecules. T cells can reject tumor due to such molecules thus eliciting immune response.

Our evidences point to a few directions, all deserving a few final remarks that we summarize in five points. First, when antigen maps are created to capture differential configurations in the two cell line scenarios, a superior connectivity appears in the HS294-T cell line vs the SKMEL-2 cell line. While some commonalities persist when considering the unconstrained gene lists, the established thresholds enable constraints that lead only for the HS294-T map to trackable and interpretable paths. These differential network signatures seem to reflect the metastatic potential of the cell lines. Therefore, by exploiting the identified antigens the connected target genes suggest that targets are better actionable in the presence of an increased metastatic potential.

Second, the expression thresholds that we adopted exert substantial effects, and expectedly constrain the systems under study by reducing the potential of exploitable target connectivity observed under unconstrained scenarios. This effect shows up by complete link depletion in the non-metastatic case. The thresholds are surely affected by the re-activation of expression levels induced by the demethylation treatment. However, an increased number of re-activated genes doesn’t necessarily means that more melanoma targets become available. Indeed these targets appear as associated to the metastatic power. In other terms, while the protein-protein interaction network maps revealed increased treatment effects in the context of non-metastatic cell line, the antigen network maps showed more actionable targets with the metastatic cell line. Target actionability in a network context adheres strictly to the property of network connectivity, and the topological measures such as degree and centrality that can be derived. Intuitively, the presence of connected target paths translates into increased chances to be able to use if not directly these targets, at least their close interactors.

Third, any current evidence must be seen in light of technological limitations, one being that the database resources here used are specific. In general, a systematic and/or comparative evaluation of putative antigens as targets of antitumor immunity is not yet available [60]. Conversely, the impact of cancer immunotherapy is constantly growing, particularly in light of the fact that the mutational load is a limited marker by itself. Whether T cell activity is the ultimate effector mechanism is something deserving further study, in association with other aspects making more complete the present cancer-immune interactome, and thus refining the “cancer immunogram” towards personalized treatments [61].

Fourth, while significant methylation appears at both promoter and gene body levels, it is quite hard to measure the influence of these values over the potential of connected targets at varying metastatic stages in view of tumor T-cell antigens. It is a topic that deserves further investigation, and here probably suffers from the incompleteness of the available data for which a clear coupled-profiles correlation is observed only at promoter level. Nonetheless, the complex regulations in the presence of differential metastatic potential have pinpointed interesting identifications that may guide the choice of candidate genes for further validation stages. Fifth and last point is a general limitation in our study. The observed patterns may provide insights into biological processes driving metastasis transition, but the limitations imposed by the scale of the experiments must be considered too. Therefore, further confirmation of the findings relative to the observed phenotypes require additional verification that can ultimately be achieved through scaled up validations and applications of biological models.

## Supporting information

S1 Tables DEG profiles and ncRNA biotypes. (XLSX)

S2 Tables Biotype classification. (XLSX)

S3 Tables Biotype annotation. (XLSX)

S4 Tables Cross-profiles (links between differential expression and differential methylation values). (XLSX)

S5 Tables Measurements of methylation levels. (XLSX)

S6 Tables DiP bio-annotations SKMEL-2. (XLSX)

S7 Tables DiP bio-annotations HS294T. (XLSX)

S8 Tables TANTIGEN results. (XLSX)

S1 Text Implementation of gls function in R. (DOCX)

S2 Text lncRNA (*lincRNome*) cross-referencing. (PDF)

S3 Text Chromatin (*DAnCER*) cross-referencing. (PDF)

S1 Fig Biological validation. (RTF)
