# Supplementary material for: Network assessment of demethylation treatment in melanoma: Differential transcriptome-methylome and antigen profile signatures
Source: PLoS One. 2018 Nov 28;13(11):e0206686. doi: 10.1371/journal.pone.0206686 (PMC6261551; doi:10.1371/journal.pone.0206686)
Supplement: S1 Text — (DOCX) [file pone.0206686.s009.docx]

**HS294 cell line**

**lm(formula = treated ~ promoter + gene, data = cell.lm.hs294)**

Residuals:

Min 1Q Median 3Q Max

-0.08896 -0.06331 -0.03516 0.00532 0.90278

Coefficients:

Estimate Std. Error t value Pr(>|t|)

(Intercept) 0.091075 0.008228 11.069 < 2e-16 ***

promoter -0.082930 0.028321 -2.928 **0.00354** **

gene -0.019403 0.036004 -0.539 0.59015

---

Signif. codes: 0 ‘***’ 0.001 ‘**’ 0.01 ‘*’ 0.05 ‘.’ 0.1 ‘ ’ 1

Residual standard error: 0.1192 on 619 degrees of freedom

Multiple R-squared: 0.02014, Adjusted R-squared: 0.01697

F-statistic: 6.361 on 2 and 619 DF, p-value: 0.001843

Correlation of Coefficients:

(Intercept) promoter

promoter -0.14

gene -0.66 -0.44

**gls(model = treated ~ promoter + gene, data = cell.lm.hs294)**

Generalized least squares fit by REML

Model: treated ~ promoter + gene

Data: cell.lm.hs294

AIC BIC logLik

-856.745 -839.0326 432.3725

Coefficients:

Value Std.Error t-value p-value

(Intercept) 0.09107515 0.00822812 11.068762 0.0000

promoter -0.08292976 0.02832146 -2.928160 **0.0035**

gene -0.01940255 0.03600404 -0.538899 0.5902

Correlation:

(Intr) promtr

promoter -0.140

gene -0.658 -0.440

Standardized residuals:

Min Q1 Med Q3 Max

-0.74643473 -0.53116008 -0.29500774 0.04462988 7.57454516

Residual standard error: 0.1191865

Degrees of freedom: 622 total; 619 residual

**SK-MEL-2 cell line**

**lm(formula = treated ~ promoter + gene, data = cell.lm.sk.mel.2)**

Residuals:

Min 1Q Median 3Q Max

-0.08913 -0.05909 -0.03724 0.00444 0.92026

Coefficients:

Estimate Std. Error t value Pr(>|t|)

(Intercept) 0.09215 0.00713 12.924 < 2e-16 ***

promoter -0.06202 0.02387 -2.598 **0.00954** **

gene -0.05533 0.04309 -1.284 0.19941

---

Signif. codes: 0 ‘***’ 0.001 ‘**’ 0.01 ‘*’ 0.05 ‘.’ 0.1 ‘ ’ 1

Residual standard error: 0.1211 on 839 degrees of freedom

Multiple R-squared: 0.01357, Adjusted R-squared: 0.01122

F-statistic: 5.772 on 2 and 839 DF, p-value: 0.003239

Correlation of Coefficients:

(Intercept) promoter

promoter -0.27

gene -0.64 -0.31

**gls(model = treated ~ promoter + gene, data = cell.lm.sk.mel.2)**

Generalized least squares fit by REML

Model: treated ~ promoter + gene

Data: cell.lm.sk.mel.2

AIC BIC logLik

-1141.286 -1122.357 574.6428

Coefficients:

Value Std.Error t-value p-value

(Intercept) 0.09215205 0.00713010 12.924368 0.0000

promoter -0.06201575 0.02387149 -2.597900 **0.0095**

gene -0.05533238 0.04308573 -1.284239 0.1994

Correlation:

(Intr) promtr

promoter -0.270

gene -0.644 -0.308

Standardized residuals:

Min Q1 Med Q3 Max

-0.73599214 -0.48790204 -0.30752404 0.03665553 7.59882789

Residual standard error: 0.1211055

Degrees of freedom: 842 total; 839 residual
