# Supplementary material for: Network assessment of demethylation treatment in melanoma: Differential transcriptome-methylome and antigen profile signatures
Source: PLoS One. 2018 Nov 28;13(11):e0206686. doi: 10.1371/journal.pone.0206686 (PMC6261551; doi:10.1371/journal.pone.0206686)
Supplement: S2 Text — (PDF) [file pone.0206686.s010.pdf]

ISTITUTO DI FISIOLOGIA CLINICA
IFC CNR

Analysis of HS294T and SK-MEL-2 cell-lines of Melanoma

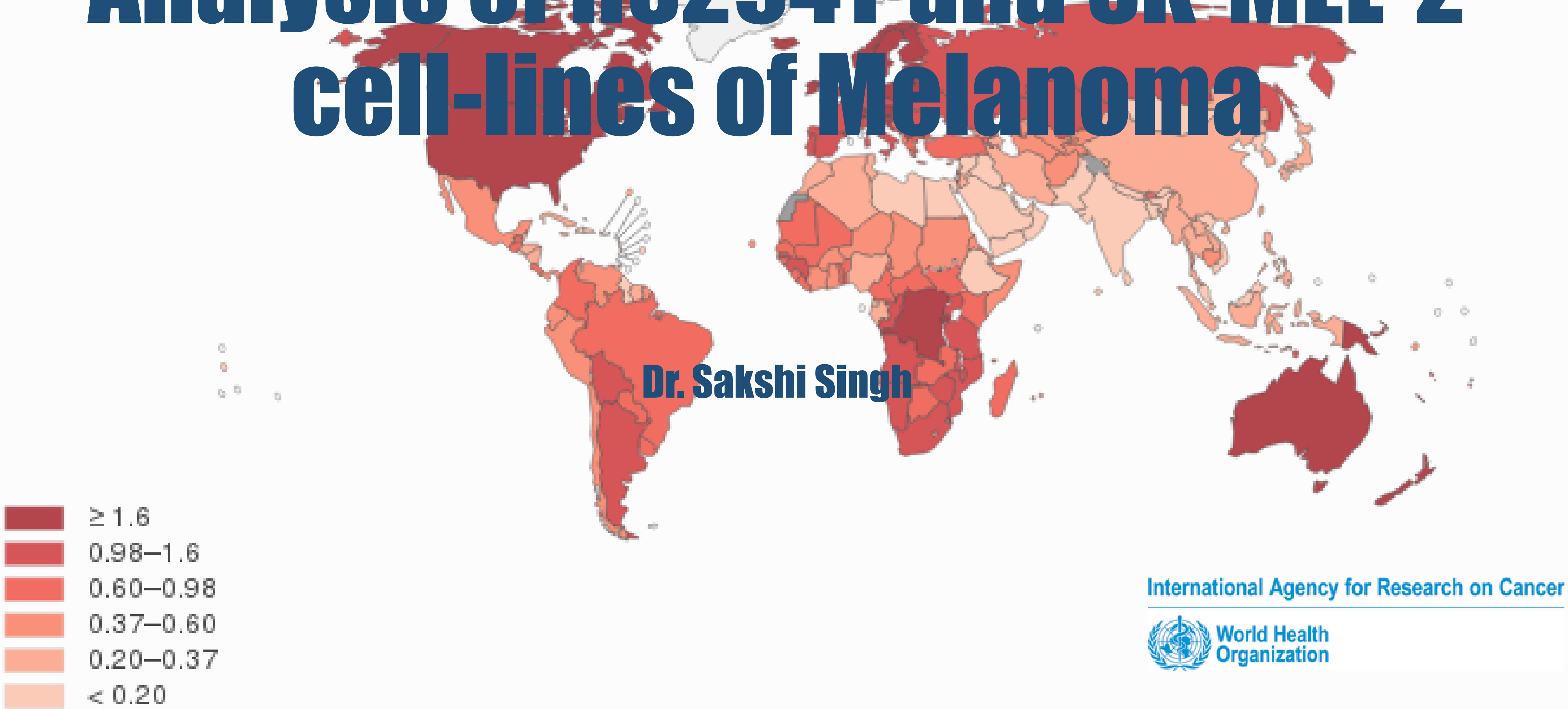

Dr. Sakshi Singh

International Agency for Research on Cancer
World Health Organization

Intersection of non coding RNAs with our experiment and lncRNome database in HS294T cell line

linc-RNA	Targets	miRNA Bindings	Transcript Type	Gene-ID
LINC00324	CLDN7, KCTD11, SOX15, EFNB3, KDM6B, ALOX12B, ALOXE3, HES7, MYH10	hsa-miR-27b-5p hsa-miR-33b-3p hsa-miR-3691-3p hsa-miR-378g hsa-miR-4476 hsa-miR-4526 hsa-miR-4652-3p hsa-miR-4779 hsa-miR-493-5p hsa-miR-5589-5p hsa-miR-574-3p hsa-miR-600 hsa-miR-9-5p	ANTISENSE	HSA-LNCG000124
LINC0327	TNFRSF19		LINC-RNA	HSA-LNCG003793
SAC-AS1	SACS		LINC-RNA	HSA-LNCG002623
TP53TG1	STEAP4		PROSCESSED-TRANSCRIPT	HSA-LNCG000155
MIAT	0		PT	HSA-LNCG001345

Target genes of LINC00324 non coding RNA that can be controlled with miRNA in HS294T cell line

ID	logFC	Gene Name	Functions
CLDN7	6.690	Claudin 7	Negative Regulation Of Cell Adhesion, Positive Regulation Of Cell Proliferation, Calcium-independent Cell-cell Adhesion Via Plasma Membrane Cell-adhesion Molecules, Negative Regulation Of Apoptotic Process, Positive Regulation Of Cell Motility,
SOX15	4.893	Sry-box 15	Negative Regulation Of Transcription From RNA Polymerase II Promoter, Positive Regulation Of Myoblast Proliferation,
ALOX12B	4.421	Arachidonate 12-lipoxygenase, 12R Type	Protein Lipidation, Positive Regulation Of MAPK Cascade, Establishment Of Skin Barrier, Positive Regulation Of Mucus Secretion
ALOXE3	2.672	Arachidonate Lipoxygenase 3	Sphingolipid Metabolic Process, Sensory Perception Of Pain, Lipoxygenase Pathway, Peroxisome Proliferator Activated Receptor Signaling Pathway, Fat Cell Differentiation, Establishment Of Skin Barrier,
HES7	2.655	Hes Family Bhlh Transcription Factor 7	Negative Regulation Of Transcription From RNA Polymerase II Promoter, Transcription, Dna-templated, Notch Signaling Pathway, Mesoderm Development, Regulation Of Somitogenesis
KCTD11	1.251	Potassium Channel Tetramerization Domain Containing 11	Negative Regulation Of Neuroblast Proliferation, Protein Ubiquitination, Negative Regulation Of Smoothened Signaling Pathway
KDM6B	1.117	Lysine Demethylase 6B	Inflammatory Response To Antigenic Stimulus, Endothelial Cell Differentiation, Positive Regulation Of Transcription From RNA Polymerase II Promoter, Mesodermal Cell Differentiation, Histone H3-K27 Demethylation,
MYH10	-1.100	Myosin Heavy Chain 10	Mitotic Cytokinesis, Exocytosis, Cell Adhesion, Cell Proliferation, Regulation Of Cell Shape
EFNB3	-1.523	Ephrin B3	Cell-cell Signaling, T Cell Costimulation, Ephrin Receptor Signaling Pathway

Summary of Target DEGs of lincRNA in SK-MEL-2 cell line (1MB distance from locus)

- There are 69 targets of non-coding RNAs in SK-MEL-2 cell line.
- Upregulated expression of KLHDC8A, the encoded protein may provide an alternative pathway for tumors to maintain aggressiveness in the absence of epidermal growth factor receptor dependence
- Marginal Zone B And B1 (MZB1) cause cell Specific Protein regulation of apoptosis, hormone-regulated chronic inflammation, affects cellular expansion and blunting insulin response in adipocytes.
- Tumor necrosis factor receptor superfamily, member 25(TNFRSF25) is an important gene as its involved in apoptosis.
- myxovirus resistance 1 (MX2) or interferon-inducible protein p78 (mouse) induces apoptosis.

Intersection of non coding RNAs with our experiment and lncRNome database in SK-MEL-2 cell line

NAME	GENE ID	TRANSCRIPT TYPE	TARGET GENES
XIST	HSA-LNCG002710	processed transcript	0
MIAT	HSA-LNCG001345	processed transcript	0
LINC00174	HSA-LNCG000129	lincRNA	0
LINC00337	HSA-LNCG001097	antisense	KCNAB2,CHD5,GPR153,HES2,ESPN,TNFRSF25,PLEKHG5
LINC00460	HSA-LNCG003989	lincRNA	EFNB2
LINC00472	HSA-LNCG003887	processed transcript	0
LINC00479	HSA-LNCG004956	processed transcript	MX2,MX1
LINC00518(2MB of distance)	HSA-LNCG000167	processed transcript	ERVFRD-1

Intersection of non coding RNAs with our experiment and lncRNome database in SK-MEL-2 cell line

- **There are only 8 non coding RNAs that are also reported in databases.**
- **Among these non-coding RNAs only one LINC00337 is having target genes within the 1Mb of distance from locus.**
- **There are 7 target gene KCNAB2, CHD5, GPR153, HES2, ESPN, TNFRSF25, PLEKHG5. Among these 6 genes are up-expressed and only 1 KCNAB2 is down-expressed.**
- **KCNAB2 promotes expression of the pore-forming alpha subunits at the cell membrane, and thereby increases channel activity and also promotes potassium channel closure via a mechanism that does not involve physical obstruction of the channel pore, Promotes KCNA4 channel closure. Modulates the functional properties of KCNA5. Enhances KCNB2 channel activity. Binds NADPH and has NADPH-dependent aldoketoreductase activity. regulating neurotransmitter release, heart rate, insulin secretion, neuronal excitability, epithelial electrolyte transport, smooth muscle contraction, and cell volume.**
- **CHD5 gene is a potential tumor suppressor gene that may play a role in the development of neuroblastoma. Tumor suppressor, it regulates the expression of genes involved in cell proliferation and differentiation. Downstream activated genes may include CDKN2A that positively regulates the p53/TP53 pathway, which in turn, prevents cell proliferation.**
- **GPR153 and HES2 are involved in transcription activity while TNRFSF25 induces apoptosis. PLEKHG5 is involved in signal transducer activity and Rho guanyl-nucleotide exchange factor activity.**
- **ESPN Plays a major role in regulating the organization, dimensions, dynamics and signaling capacities of the actin filament-rich, microvillus-type specializations that mediate sensory transduction in various mechanosensory and chemosensory cells.**
