# Supplementary material for: Network assessment of demethylation treatment in melanoma: Differential transcriptome-methylome and antigen profile signatures
Source: PLoS One. 2018 Nov 28;13(11):e0206686. doi: 10.1371/journal.pone.0206686 (PMC6261551; doi:10.1371/journal.pone.0206686)
Supplement: S3 Text — (PDF) [file pone.0206686.s011.pdf]

IFC CNR Istituto di Fisiologia Clinica

Analysis of HS294T and SK-MEL-2 cell-lines of Melanoma

Dr. Sakshi Singh

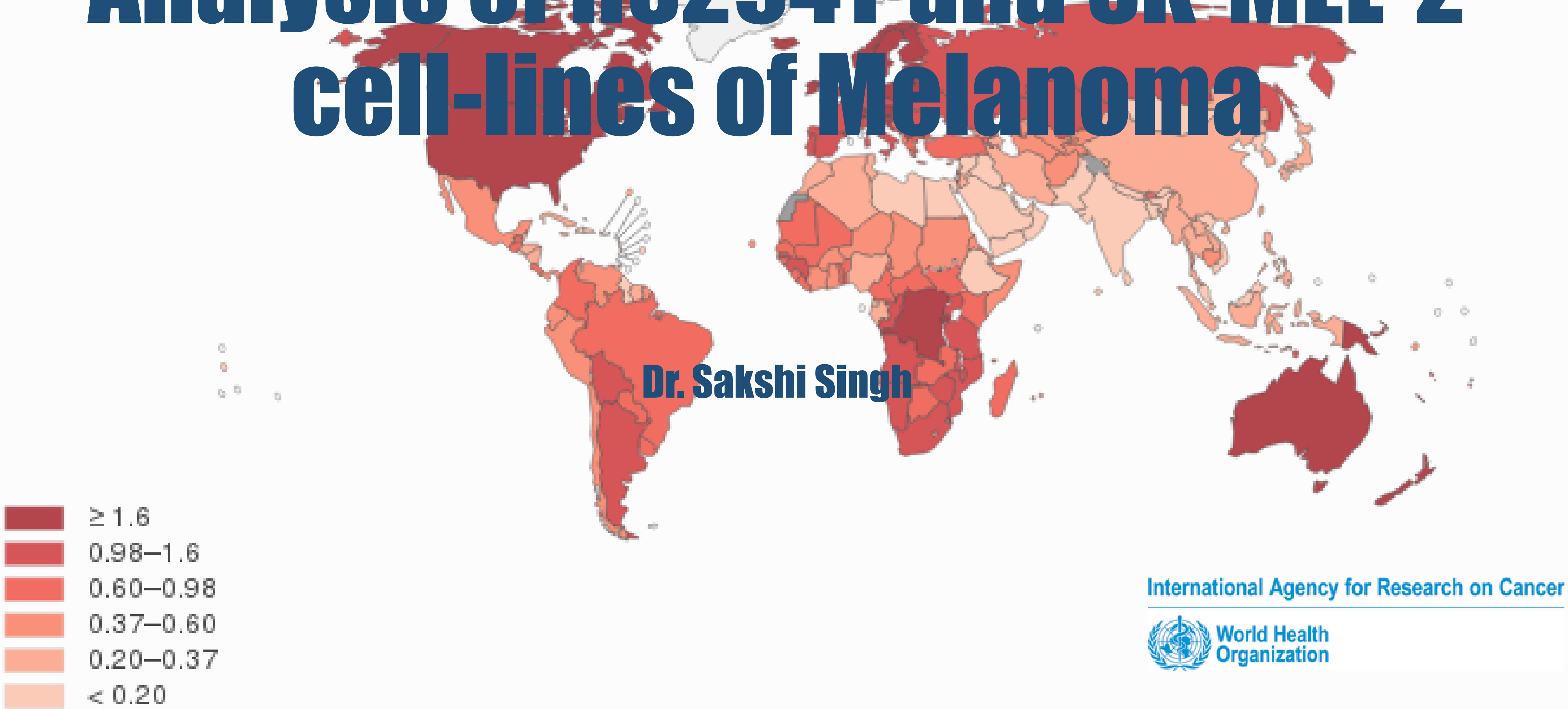

International Agency for Research on Cancer

World Health Organization

Genes CONFIRMED In Chromatin Modeling HS294T cell line from DAnCER (Disease Annotated Chromatin Epigenetics Resource)-1

ID	logFC	Gene Name	Function	ID	logFC	Gene Name	Function
H2AFB2	8.83	H2A histone family, member B2	DNA packaging,chromatin organization	HIST1H2BJ	3.043	Histone cluster 1, h2bj	DNA packaging, chromatin organization
CTCFL	8.672	CCCTC-binding factor (zinc finger protein)-like	DNA metabolic process, DNA modification	SERPIND1	2.828	Serpin peptidase inhibitor, clade D (heparin cofactor), member 1	Chemotaxis, blood coagulation, hemostasis, response to chemical stimulus
SYCP3	5.631	Synaptonemal complex protein 3	M phase, reproductive developmental process, DNA packaging	HIST1H4H	2.695	Histone cluster 1, h4h	Regulation of immune system process, DNA packaging, negative regulation of myeloid cell differentiation
AURKC	4.477	Aurora kinase C	cytokinesis, protein modification process	HIST1H2AE	2.655	Histone cluster 1, h2ae	DNA packaging, chromatin organization
HIST1H2BG	3.665	Histone cluster 1, h2bg	DNA packaging, chromatin organization	HIST2H2AA3	2.443	Histone cluster 2, h2aa3	DNA packaging, chromatin organization
HIST1H3D	3.146	Histone cluster 2, h3d	DNA packaging, chromatin organization	HIST2H2AA4	2.443	Histone cluster 2, h2aa4	DNA packaging, chromatin organization

Genes CONFIRMED In Chromatin Modeling HS294T cell line from DAnCER (Disease Annotated Chromatin Epigenetics Resource)-2

ID	logFC	Gene Name	Function	ID	logFC	Gene Name	Function
HIST1H2BC	2.441	Histone cluster 1, h2bc	DNA packaging, chromatin organization	HIST1H2BD	1.555	Histone cluster 1, h2bd	DNA packaging, chromatin organization
HIST3H2A	1.804	Histone cluster 3, h2a	DNA packaging, chromatin organization	NKX3-1	1.546	NK3 homeobox 1	Nucleobase metabolic process, transcription
H2AFJ	1.795	H2A histone family, member J	DNA packaging, chromatin organization	HIST1H2BK	1.499	Histone cluster 1, h2bk	DNA packaging, chromatin organization
TUBA4A	1.75	Tubulin, alpha 4a	Protein complex assembly, microtubule-based movement	KAT2B	-1.509	K(lysine) acetyltransferase 2B	Chromatin organization, n-terminal protein amino acid acetylation
HIST1H1C	1.673	Histone cluster 1, h1c	DNA packaging, chromatin organization	MGAM	-1.723	Maltase-glucoamylase (alpha-glucosidase)	Polysaccharide catabolic process
PADI4	1.624	Peptidyl arginine deiminase, type IV	Citrulline metabolic process	ZEB2	-1.77	Zinc finger e-box binding homeobox 2	Ameboidal cell migration, regulation of protein amino acid phosphorylation
MUC1	1.561	Mucin 1, cell surface associated	Reproduction, response to vitamin A, response to chemical stimulus	LMNB1	-1.906	Lamin B1	Regulation of activated pak-2p34 by proteasome mediated degradation9

Genes CONFIRMED In Chromatin Modeling HS294T cell line from DAnCER (Disease Annotated Chromatin Epigenetics Resource)

- **I found 40 genes that are being also reported in DAnCER to be involved in chromatin modelling in treated HS294T melanoma cell-line with demethylating drug.**
- **Many of these genes are highly differentially expressed 22 genes highly up-expressed while 4 highly down-expressed (-1.5> logFC >+1.5).**
- **The genes like H2AFB2, CTCFL, SYCP3, AURKC, HIST1H2BG, HIST1H3D, HIST1H2BJ, SERPIND1 are among the highly up-expressed genes.**
- **KAT2B, ZEB2, MGAM, LMNB1 are highly down expressed genes.**
- **Other then being involved in DNA packaging and chromatin modelling, these genes are also known to be playing role in cancer, for example SERPIN family is involved in metastasis of cancer.**
- **Among down expressed genes, LMNB1 is regulate PAK-2p34 by protease mediated degradation, MGAM is involved in metabolism and immune system, ZEB2 is involved in TGF beta signalling pathway and microRNAs, KAT2B play role in SMAD2/3 signalling.**

Genes CONFIRMED In Chromatin Modeling SK-MEL-2 cell line from DAnCER (Disease Annotated Chromatin Epigenetics Resource)-1

ID	logFC	Gene Name	Functions	ID	logFC	Gene Name	Functions
H2AFB2	6.180	H2A histone family member B2	DNA binding, protein heterodimerization activity,	CPA4	2.791	carboxypeptidase A4	metallocarboxypeptidase activity
SYCP3	6.129	synaptonemal complex protein 3	DNA binding	CTCFL	2.538	CCCTC-binding factor like	RNA polymerase II core promoter proximal region sequence-specific DNA binding
TUBA4A	3.868	tubulin alpha 4a	GTPase activity	HIST1H2BC	2.184	histone cluster 1 H2B family member c	DNA binding
HIST1H2AG	3.583	histone cluster 1 H2A family member g	DNA binding	HIST2H2AA3	2.182	histone cluster 2 H2A family member a3	DNA binding
SERPIND1	3.527	serpin family D member 1	serine-type endopeptidase inhibitor activity	HIST2H2AA4	2.182	histone cluster 2 H2A family member a4	DNA binding
HIST1H2BG	3.322	histone cluster 1 H2B family member g	DNA binding	TLE4	2.139	transducin like enhancer of split 4	chromatin binding
HIST1H4H	3.182	histone cluster 1 H4 family member h	DNA binding	HIST1H2BJ	2.088	histone cluster 1 H2B family member j	DNA binding
HIST1H2AI	2.997	histone cluster 1 H2A family member i	DNA binding	PCSK4	2.048	proprotein convertase subtilisin/kexin type 4	serine-type endopeptidase activity
MUC1	2.937	mucin 1, cell surface associated	RNA polymerase II core promoter proximal region sequence-specific DNA binding, p53 binding	H1F0	2.019	H1 histone family member 0	chromatin DNA binding

Genes CONFIRMED In Chromatin Modeling SK-MEL-2 cell line from DAnCER (Disease Annotated Chromatin Epigenetics Resource)-2

ID	logFC	Gene Name	Functions	ID	logFC	Gene Name	Functions
HIST1H3H	1.766	histone cluster 1 H3 family member h	nucleosomal DNA binding	HIST1H1 C	1.061	histone cluster 1 H1 family member c	chromatin DNA binding
HIST1H3D	1.698	histone cluster 1 H3 family member d	nucleosomal DNA binding	CDK2AP1	-1.066	cyclin dependent kinase 2 associated protein 1	DNA polymerase binding
NKX3-1	1.478	NK3 homeobox 1	transcription factor activity, MADS box domain binding,	NPM1	-1.097	nucleophosmin	transcription coactivator activity
HIST1H2BD	1.435	histone cluster 1 H2B family member d	DNA binding	SKI	-1.107	SKI proto-oncogene	transcriptional repressor activity
HMGA1	1.375	high mobility group AT-hook 1	transcriptional activator activity, RNA polymerase II distal enhancer sequence-specific binding	SAP30	-1.181	Sin3A associated protein 30	transcription corepressor activity
HIST2H2BE	1.320	histone cluster 2 H2B family member e	protein heterodimerization activity	H1FX	-1.199	H1 histone family member X	cadherin binding involved in cell-cell adhesion
BCL6	1.277	B-cell CLL/lymphoma 6	RNA polymerase II regulatory region sequence-specific DNA binding	PHF2	-1.220	PHD finger protein 2	transcription coactivator activity,methylated histone binding
SIRT7	1.191	sirtuin 7	chromatin binding, NAD-dependent histone deacetylase activity (H3-K18 specific)	LMNB1	-1.284	lamin B1	phospholipase binding

Genes CONFIRMED In Chromatin Modeling SK-MEL-2 cell line from DAnCER (Disease Annotated Chromatin Epigenetics Resource)

- **I found 34 genes that are being also reported in DAnCER to be involved in chromatin modelling in treated SK-MEL-2 melanoma cell-line with demethylating drug.**
- **Among these 34 genes 19 genes are recurrent with HS294T cell-line.**
- **These 15 genes exclusively involved in chromatin modeling in SK-MEL-2 are SIRT7, PCSK4, H1F0, BCL6, SAP30, HIST1H3D, TLE4 etc.**
- **SIRT7 has been found to involved in many processes like aging, cancer and circadian rhythms also.**
- **BCL6 is involved in immune system and is a transcriptional repressor.**

Putative Chromatin Modeling In Melanoma And Skin Neoplasm in HS294T cell line DAnCER (Disease Annotated Chromatin Epigenetics Resource)

Melanoma Gene ID	Gene Name	logFC	Functions
SOX10	SRY (Sex Determining Region Y)-Box 12	-1.094	ERK Signaling, Neural Stem Cell Differentiation Pathways, Lineage-specific Markers
POU3F2	POU Class 3 Homeobox 2	-1.549	Somatic Malignant Melanoma, transcription factor activity

Skin Neoplasm Gene ID	Gene Name	logFC	Functions
SOX10	SRY (Sex Determining Region Y)-Box 12	-1.094	ERK Signaling, Neural Stem Cell Differentiation Pathways, Lineage-specific Markers
GLI2	Glioma-Associated Oncogene Family Zinc Finger 2	-1.316	Pathways in cancer, Wnt Signaling Pathways
IRF4	Interferon Regulatory Factor 4	-1.833	Interferon gamma signaling and B cell receptor signaling pathway

*A L Turinsky, et al. **DAnCER: Disease-Annotated Chromatin Epigenetics Resource.** *Nucleic Acids Research (2011) 39(suppl 1): D889-D894*

Putative Chromatin Modeling In Melanoma And Skin Neoplasm in SK-MEL-2 cell line DAnCER (Disease Annotated Chromatin Epigenetics Resource)

Melanoma Gene ID	Gene Names	logFC	Functions
MC1R	Melanocortin 1 Receptor	-1.189	G-protein coupled receptor activity and hormone binding
POU3F2	POU Class 3 Homeobox 2	-1.209	Somatic Malignant Melanoma, transcription factor activity

Gene ID	Gene Names	logFC	Disease	Functions
NOS3	Nitric Oxide Synthase 3	5.174	Skin Disease	VEGF Signaling Pathway and Activation of cAMP-Dependent PKA
ACTA2	Actin, Alpha 2, Smooth Muscle, Aorta	4.862	Vascular Skin Disease	EPHA forward signaling and Arrhythmogenic right ventricular cardiomyopathy (ARVC)
GLI1	GLI Family Zinc Finger 1	2.036	Skin Neoplasm	Activation of cAMP-Dependent PKA, Pathways in cancer
MC1R	Melanocortin 1 Receptor	-1.189	Skin Neoplasm	G-protein coupled receptor activity and hormone binding

*A L Turinsky, et al. **DAnCER: Disease-Annotated Chromatin Epigenetics Resource.** *Nucleic Acids Research (2011) 39(suppl 1): D889-D894*
