# Supplementary material for: Network assessment of demethylation treatment in melanoma: Differential transcriptome-methylome and antigen profile signatures
Source: PLoS One. 2018 Nov 28;13(11):e0206686. doi: 10.1371/journal.pone.0206686 (PMC6261551; doi:10.1371/journal.pone.0206686)
Supplement: S1 Fig — (RTF) [file pone.0206686.s012.rtf]

Figure 7: Analysis of apoptotic markers in HS294T and SKMEL-2 for untreated cells (ctrl) and cells treated with 10 M 5-Aza-2'-Deoxycytidine for 72h (DAC). Densitometric analysis of PARP1 and lamin A immunoblotted bands is reported in the graphs. Data are reported as mean ± sd. The statistical significance threshold was set at p-value < 0.05.
